# Autoimmune disease: a view of epigenetics and therapeutic targeting

**DOI:** 10.3389/fimmu.2024.1482728

**Published:** 2024-11-13

**Authors:** Siqi Mu, Wanrong Wang, Qiuyu Liu, Naiyu Ke, Hao Li, Feiyang Sun, Jiali Zhang, Zhengwei Zhu

**Affiliations:** ^1^ Department of Dermatology, The First Affiliated Hospital of Anhui Medical University, Hefei, Anhui, China; ^2^ Key Laboratory of Dermatology (Anhui Medical University), Ministry of Education, Hefei, Anhui, China; ^3^ Department of Skin Genetics, Anhui Province Laboratory of Inflammation and Immune Mediated Diseases, Hefei, Anhui, China; ^4^ Department of Dermatology, Shannan People's Hospital, Shannan, China; ^5^ First Clinical Medical College, Anhui Medical University, Hefei, Anhui, China; ^6^ Department of Respiratory and Critical Care Medicine, First Affiliated Hospital of Anhui Medical University, Hefei, Anhui, China; ^7^ Department of Pharmacology, Basic Medical College, Anhui Medical University, Hefei, Anhui, China; ^8^ Department of Ophthalmology, First Affiliated Hospital of Anhui Medical University, Hefei, Anhui, China; ^9^ Department of Urology, First Affiliated Hospital of Anhui Medical University, Hefei, Anhui, China

**Keywords:** autoimmune disease, epigenetic, systemic lupus erythematosus, DNA methylation, histone modification, RNA modification

## Abstract

Autoimmune diseases comprise a large group of conditions characterized by a complex pathogenesis and significant heterogeneity in their clinical manifestations. Advances in sequencing technology have revealed that in addition to genetic susceptibility, various epigenetic mechanisms including DNA methylation and histone modification play critical roles in disease development. The emerging field of epigenetics has provided new perspectives on the pathogenesis and development of autoimmune diseases. Aberrant epigenetic modifications can be used as biomarkers for disease diagnosis and prognosis. Exploration of human epigenetic profiles revealed that patients with autoimmune diseases exhibit markedly altered DNA methylation profiles compared with healthy individuals. Targeted cutting-edge epigenetic therapies are emerging. For example, DNA methylation inhibitors can rectify methylation dysregulation and relieve patients. Histone deacetylase inhibitors such as vorinostat can affect chromatin accessibility and further regulate gene expression, and have been used in treating hematological malignancies. Epigenetic therapies have opened new avenues for the precise treatment of autoimmune diseases and offer new opportunities for improved therapeutic outcomes. Our review can aid in comprehensively elucidation of the mechanisms of autoimmune diseases and development of new targeted therapies that ultimately benefit patients with these conditions.

## 1 Introduction

Autoimmune diseases represent a diverse group of conditions characterized by an imbalanced immune system that abnormally attacks the cells and tissues of the body, loss of immune tolerance to autoantigens, and impaired self-nonself discrimination. This dysfunction leads to excessive proliferation and activation of autoreactive T- or B-cells, resulting in the unintentional targeting of normal tissues (1). The establishment of immune tolerance involves several different mechanisms and interactions, resulting in disease-specific heterogeneity in autoimmune diseases (2). The clinical symptoms of autoimmune diseases are highly heterogeneous and related to the specific site of involvement (**Table 1**); accordingly, they can be categorized into organ-specific and systemic autoimmune diseases (42). However, most of these diseases have similar manifestations, such as the tissue inflammatory response, which is caused by local immune cell infiltration and inflammatory factors (43). In certain diseases, the occurrence of lesions and damage can be triggered by autoantibodies secreted by plasma cells or directly mediated by pathogenic T-cells. Therefore, most autoimmune diseases are hypothesized to have a shared etiology and pathogenesis (44). For instance, genetic factors exert substantial influence on the pathogenesis of autoimmune diseases, and genome-wide association studies (GWAS) are powerful tools for studying genetic susceptibility to disease. However, the technique is limited to analysis at the genetic level, and interactions between genes and downstream factors are not adequately considered; thus, the pathogenesis and regression of autoimmune diseases cannot be comprehensively revealed (45).

**Table 1:** Specific antibodies, clinical manifestations, and treatment of autoimmune diseases.

	Disease	Autoantibody	Treatment	References
Rheumatic autoimmune diseases	Systemic lupus erythematosus (SLE)	Anti-nuclear antibodies (ANAs): anti-double-stranded DNA antibody, antiphospholipid antibody, anti-Sm antibody	Nonsteroidal anti-inflammatory drugs (NSAIDs), Glucocorticoids, Hydroxychloroquine, Belimumab, Rituximab, Anifrolumab	(3, 4)
	Rheumatoid arthritis (RA)	Rheumatoid factor (RF), anti-citrullinated protein antibodies (ACPAs)	Disease-modifying anti-rheumatic drugs (DMARDs): methotrexate, hydroxychloroquine; JAK inhibitors: Tofacitinib, Baricitinib; TNF inhibitors: Adalimumab, Infliximab; Anti-IL-6: Tocilizumab, Sarilumab	(5, 6)
	Sjögren’s syndrome (SS)	ANAs; anti-ribonucleoprotein complexes Ro/SSA and La/SSB antibodies; RF	Glucocorticoids, hydroxychloroquine, anti BAFF antibody belimumab	(7, 8)
	Antiphospholipid syndrome (APS)	Anti-phospholipid antibody, anti-β2 glycoprotein-I antibody, anticardiolipin antibodies	Anti-coagulants, Vitamin K antagonists (VKAs), Aspirin, Hydroxychloroquine	(9, 10)
	Systemic sclerosis (SSc)	ANAs, anti-topoisomerase I (Scl-70), anti-nucleolar antibody, anti-RNA polymerase I/III antibody	Mycophenolate, cyclophosphamide, Tocilizumab, Rituximab	(11, 12)
Central nervous system	Multiple sclerosis (MS)	Intrathecal oligoclonal IgM antibodies against myelin lipids, preferentially phosphatidylcholine; anti-Sperm-associated antigen 16 (SPAG16) antibodies	Ocrelizumab, interferons, glatiramer acetate, dimethyl fumarate	(13, 14)
	Guillain–Barré syndrome (GBS) subtype: acute motor axonal neuropathy (AMAN)	Anti-ganglioside antibodies: anti-GM1a, GM1b, GD1a antibodies	Intravenous immunoglobulin (IVIg) and plasma exchange	(15, 16)
	Autoimmune limbic encephalitis (ALE)	Anti-leucine-rich glioma inactivated 1 antibody, anti-γ-aminobutyric acid B receptor antibody, anti-Hu antibody	Intravenous immunoglobulin (IVIG), plasma exchange (PLEX), rituximab	(17, 18)
	Anti-NMDA receptor encephalitis	Anti-GluN1 subunit of the NMDA receptor antibody	Steroids, intravenous immunoglobulins (IVIG), or plasma exchange (PLEX)	(19)
Endocrine system	Type 1 diabetes mellitus	Antibodies against specific β-cell proteins, including insulin, glutamate decarboxylase, islet antigen 2, zinc transporter 8, and tetraspanin-7	Insulin, pramlintide, metformin, glucagon-like peptide-1 receptor agonists	(20, 21)
	Autoimmune Addison disease	Anti-adrenal cortex autoantibodies (ACAs): antibodies directed against steroid 21-hydroxylase	Corticosteroid replacement	(22)
Hematologic system	Autoimmune hemolytic anemia (AIHA)	Anti-RBC antigens antibodies: Warm AIHA IgG; cold AIHA IgM; Mixed-type AIHA (mAIHA) IgG, IgM; Paroxysmal cold hemoglobinuria (PCH) IgG	Glucocorticoids, splenectomy, anti-CD20 mAb	(23)
Skin	Vitiligo	Anti-epidermal melanocytes antibody	UV Treatments, Topical corticosteroids, Calcineurin inhibitors	(24, 25)
	Pemphigus	Anti-Desmoglein 1/3 autoantibody	Corticosteroids, immunosuppressant drugs	(26)
	Epidermolysis bullosa acquisita (EBA)	Anti- collagen VII antibody	Neutrophil targeting therapies, conventional Immunosuppressives, intravenous immunoglobulin and rituximab	(27, 28)
Digestive system	Primary biliary cirrhosis (PBC)	Anti-mitochondrial antibodies (AMAs), Anti-Sp100 and anti-gp210 antibodies	Ursodeoxycholic acid (UDCA)	(29, 30)
	Coeliac disease	Anti-transglutaminase 2 (TG2) and anti-endomysial (EMA) antibodies	A gluten-free diet, administration of probiotics.	(31, 32)
	Crohn’s disease	Antimicrobial antibodies: anti-Saccharomyces cerevisiae antibody (ASCA)	5-aminosalicylate, corticosteroids, anti-TNF: Infliximab, adalimumab	(33, 34)
	Autoimmune gastritis (AIG)	Anti-parietal cell antibody, anti-H+/K+ ATPase antibody, anti-intrinsic factor antibody	Intramuscular cobalamin, Iron and vitamin B12 supplementation	(35, 36)
Muscle	Myasthenia gravis (MG)	Anti-acetylcholine receptor (AChR) antibody; Anti-muscle-specific kinase antibody	Pyridostigmine, immunosuppressive medication	(37, 38)
	Lambert-Eaton myasthenic syndrome (LEMS)	Anti-P/Q-type Voltage-gated calcium channel (VGCC) antibody	3,4-diaminopyridine potassium channel blocker	(39)
Thyroid gland	Graves’ diseases (GD)	Anti-thyrotropin receptor antibody (TRAb), anti-thyroid stimulating antibody (TSAb)	Methimazole, propylthiouracil, thyroidectomy	(40)
	Hashimoto’s thyroiditis	Anti-thyroperoxidase antibody (TPOAb), anti-thyroglobulin antibody (TgAb)	Levothyroxine, glucocorticoids	(41)

**Figure 1:**
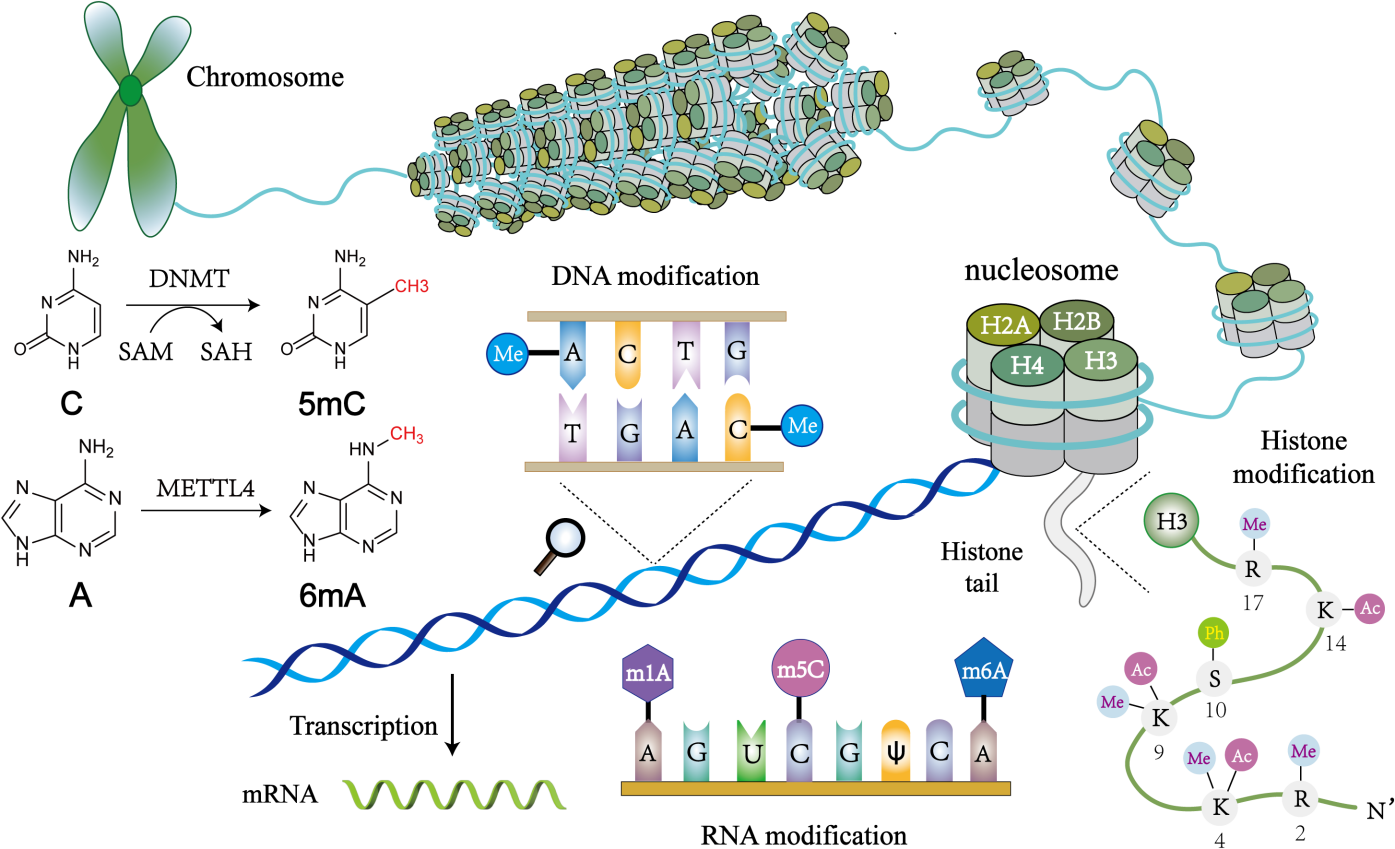
An overview of epigenetic mechanisms. The basic unit of chromatin is the nucleosome. Two molecules of H2A, H2B, H3, and H4 form a histone octamer, and DNA molecules are coiled around the histone octamer to form the nucleosome’s core particles. DNA methylation is mediated by the enzyme DNA methyltransferase (DNMT), which transfers methyl groups to adenine (A), guanine (G), or cytosine (C) by using S-adenosyl methionine (SAM) as a substrate. Histone modifications include acetylation, methylation, phosphorylation and so on, and they mainly occur on lysine (K), arginine (R) and serine (S). RNA modifications are similar to DNA modifications, including m1A, m6A and many other types. Chemical structures are prepared using ChemDraw.

Although most autoimmune diseases are inextricably linked to genetic factors, recent advance in epigenetics has greatly enriched our understanding of their pathogenesis, and provided a comprehensive review of the pathogenesis of the mechanisms involved. Epigenetics elucidates the unconventional changes in gene expression that ultimately lead to differences in phenotypes without altering DNA sequences (46). The core of the epigenetic regulatory mechanism is a variety of reversible covalent modifications of nucleic acids and histones in the presence of multiple chemical modifying enzymes, such as DNA/RNA methylation and histone acetylation (**Figure 1**, **Table 2**). Meanwhile, these modifications can induce sophisticated crosstalk for regulating gene expression (49). In addition, the mechanisms of epigenetic regulation also include chromatin remodeling and non-coding RNAs, the former uses chromatin remodeling complexes to regulate the denseness or looseness of chromatin structure, thereby affecting the binding of transcription factors to control gene expression (58). Although non-coding RNAs are unable to translate proteins, they have an essential role in epigenetics by coordinating DNA methylation, chromatin structure remodeling, and histone chemical modification (59).

**Table 2:** Comparison of DNA, RNA, and histone modifications.

	Writer	Eraser	Reader	Types of modification	Site of modification	Biological effect	References
DNA modification	DNMT1, DNMT3A and DNMT3B	TET1/2/3	MBD, MeCP2	5mC, 6mA, 7mG	Genome DNA, mitochondrial DNA	DNA methylation at gene promoter is associated with transcriptional silencing and it mediates chromatin inaccessibility	(47–50)
RNA modification	METTL3, METTL14, WTAP, KIAA1492	FTO, ALKBH5	YTHDF1/2/3, YTHDC1/2, IGF2BP1/2/3, HNRNP protein family	m6A, m5C, m7G, Pseudouridine	messenger RNA, ribosomal RNA, transfer RNA, non-coding RNA	m6A regulates various post-transcriptional aspects of the mRNA lifecycle, including RNA degradation, processing, splicing, nuclear export, mRNA translation, and also regulates gene expression	(49, 51, 52)
Histone modification	HMT, HAT	HDM, HDAC	Chromodomain, PHD fingers, bromodomain proteins	Methylation, Acetylation, Phosphorylation, Ubiquitination	H2A, H2B, H3, H4	Transcriptional activation: H3K4me3, H3K4ac. Transcriptional repression: H3K27me3.	(53–57)

DNMT, DNA methyltransferase; TET, Ten-eleven translocation; MBD, methyl-CpG-binding domain; MeCP, methyl-CpG binding protein; HDAC, histone deacetylase; METTL, Methyltransferase like; WTAP, Wilms tumor 1-associating protein; FTO, fat-mass and obesity-associated protein; ALKBH, AlkB homologue; YTHDF, YTH domain family; YTHDC, YTH domain-containing protein; IGF2BP, Insulin-like growth factor-2 mRNA-binding proteins; HNRNP, heterogeneous nuclear ribonucleoprotein; HMT, histone methyltransferase; HAT, histone acetyltransferases; HDM, histone demethylase; HDAC, histone deacetylase; PHD, plant homeodomain.

With the advancement of detection technologies over the past few decades, knowledge and research on epigenetic mechanisms have progressively deepened, and an increasing body of evidence has shown that epigenetic mechanisms positively regulate the growth and aging of people and are closely linked to a wide variety of diseases, especially autoimmune diseases (60). Epigenetic modifications are highly dynamic systems that constantly change in response to environmental factors and organism development (61). Notably, epigenetic modifications are reversible processes mediated by a variety of enzymes, exhibiting specificity to certain cells and diseases, consistent with the fact that autoimmune diseases share similar pathogenesis but present with distinct clinical manifestations. Therefore, epigenetic modifications are promising biomarkers for disease diagnosis (62). The reversal of aberrant modifications through the use of targeted drugs has enormous potential for treating autoimmune diseases. Epigenetic drug research is an emerging field in the treatment of diseases at the level of gene regulation that opens a new path for precision medicine (63). Globally, the incidence of autoimmune diseases persists at elevated levels coupled with the absence of efficient therapeutic interventions with minimum side effects. Conventional nonsteroidal anti-inflammatory drugs (NSAIDs) can only alleviate the symptoms, while long-term use of immunosuppressive drugs compromises the immune function of patients (64). Therefore, it is essential to explore new research directions to comprehensively understand the mechanisms of autoimmune diseases and develop new targeted therapies, ultimately benefiting patients with these conditions.

In this review, we summarize the major mechanisms of autoimmune diseases and epigenetics, emphasizing the close association of epigenetic modifications with autoimmune diseases and providing novel perspectives on leveraging epigenetics for the diagnosis and treatment of diseases.

## 2 The mechanisms of autoimmune disease

### 2.1 Potential pathogenesis of autoimmune diseases

Many years ago, it was observed that autoimmune diseases had a tendency for family inheritance, with their pathogenesis primarily driven by genetic factors. GWAS provides a powerful tool for investigating disease susceptibility genetically, but this technique is limited to genetic analysis and is unable to comprehensively reveal the pathogenesis of autoimmune diseases (45). A deeper exploration of disease mechanisms has highlighted the indispensability of environmental and epigenetic factors in autoimmune disease development. Epigenetics has emerged as a prominent research topic in this field, not only bridging the gap between environmental and genetic factors but also acting as an independent factor that induces or exacerbates autoimmune diseases (65). However, the current accumulated knowledge is still unable to fully explain the heterogeneity of the disease; therefore, finding new research directions is necessary to comprehensively sort out their mechanisms.

Among the genetically driven autoimmune diseases, immunodysregulation polyendocrinopathy enteropathy X-linked (IPEX) syndrome is a classic example of a monogenic autoimmune disease in which the mutated forkhead box P3 *(FOXP3)* gene disrupts the transcription of Treg signature genes, such as interleukin 2 receptor subunit alpha (IL-2RA) and cytotoxic T-lymphocyte associated protein 4 (CTLA4), thus affecting the development and function of Tregs and ultimately resulting in several autoimmune disorders, such as type 1 diabetes mellitus (T1D) and severe bowel disease (66). Mutations in the AIRE gene impede the expression of the tissue-specific antigen by medullary thymic epithelial cells (mTECs), resulting in the compromised clearance of autoreactive T-cells or induction of Treg production, which in turn triggers a severe multiorgan damaging autoimmune disease, namely autoimmune polyendocrine syndrome type 1 (APS-1) (67). In addition, various environmental risk factors, including drugs, viral infections, and ultraviolet radiation, can influence the pathogenesis of autoimmune diseases by affecting epigenetic modifications. One hypothesis regarding the mechanism by which air pollution contributes to autoimmune diseases is that it can enhance systemic inflammatory and autoimmune responses through a variety of mechanisms (68). For instance, various infectious factors, particularly viruses, contribute to systemic lupus erythematosus (SLE) pathogenesis through multiple mechanisms. One such mechanism is molecular mimicry, which refers to the use of exogenous antigens that resemble autoantigens in their sequence or structure, thereby activating autoreactive T- or B-cells to produce autoantibodies, resulting in tissue and organ damage. A typical example is the crossreactivity between Epstein-Barr virus (EBV) nuclear antigens and host autoantigens (69). Moreover, CD8+ T-cells of patients with SLE exhibit an impaired immune response to EBV, leading to a significant increase in the EBV viral load in patients, where the latent viruses cause persistent infections and constantly stimulate the immune system to continuously produce antibodies and circulating immune complexes, ultimately exacerbating the disease (70).

Numerous relevant factors and pathological mechanisms are believed to contribute to the onset of autoimmune diseases; however, no specific theory can perfectly explain the underlying mechanisms. Therefore, comprehensively elucidating the genetic, environmental, and epigenetic factors related to autoimmune diseases is imperative. Notably, the interaction among these factors in mediating disease development is particularly important for elucidating their pathogenesis. This knowledge will enable the identification of risk factors and formulation of preventive interventions targeting susceptible populations, ultimately reducing the disease burden.

### 2.2 Dysfunctional immune cells in autoimmune diseases

**Figure 2:**
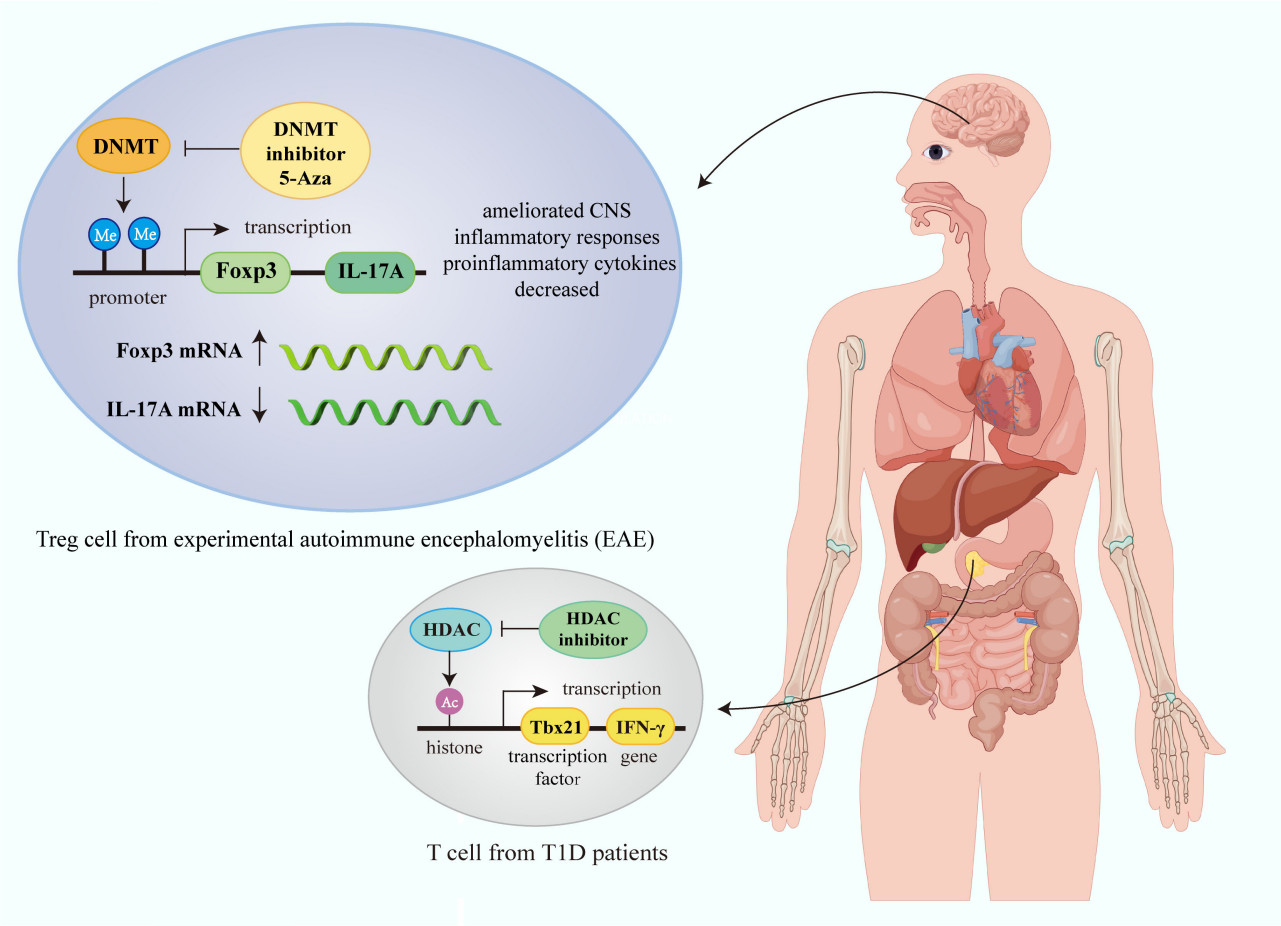
Epigenetic modification of immune cells in autoimmune diseases. Gene transcription in immune cells in autoimmune diseases is affected by epigenetic mechanisms such as histone modification and DNA methylation. In Treg cell from experimental autoimmune encephalomyelitis (EAE), DNMT inhibitor 5-Aza can promote Foxp3 transcription, and inhibit IL-17A transcription, thus ameliorating CNS inflammatory responses and decreasing proinflammatory cytokines. HDAC inhibitor can increase histone acetylation modification and promote IFN-γ transcription in T cell in T1D. (Figure created by Figdraw, ID: TUIUU9ee14).

Autoimmune diseases arise from a malfunctioning immune system and disrupted immune tolerance mechanisms, resulting in an immune response in which autoreactive lymphocytes target self-antigens and cause damage to the body (1). Under normal circumstances, host immune homeostasis is maintained by both the innate and adaptive immune systems, thus preventing the occurrence of autoimmune diseases (71). However, in patients with autoimmune diseases, the immune system exhibits abnormalities such as heightened activation of immune cells, alterations in their function and phenotype, and aberrant expression of various immune molecules, including complement, antibodies, and cytokines. This pathological state results in an inflammatory response and tissue damage, ultimately contributing to the progression of autoimmune diseases (**Figure 2**) (72).

Among the various immune cells, T- and B-lymphocytes are the most prominent mediators of autoimmunity that contribute to tissue damage, with aberrant alterations in their number and function being key mechanisms driving autoimmune diseases (73, 74). Autoreactive B-cells not only cause tissue and organ damage and exacerbate inflammatory responses through the secretion of autoantibodies and inflammatory factors but also further aggravate disease severity by presenting antigens and expressing costimulatory molecules to activated T-cells (75). B10 cells suppress immune responses by secreting interleukin 10 (IL-10); however, their number and function are dysregulated in rheumatoid arthritis (RA), as tumor necrosis factor-alpha (TNF-α) downregulates IL-10 and upregulates IFN-γ and IL-17A, inducing a proinflammatory B10 cell phenotype, leading to disease deterioration (76). CD4+CD25+forkhead box P3 (Foxp3)+ Tregs have potent immunosuppressive functions and contribute to immune tolerance maintenance and the control of autoimmune disease development. Abnormalities in the number and function of Tregs have been observed in many types of autoimmune disorders (77). Most studies demonstrated that the proportion of Tregs in SLE patients decreased with the increase in disease activity (78, 79); however, some studies have found that the number of Tregs in organ-specific autoimmune diseases increases, potentially depending on the clinical disease activity, further highlighting the great heterogeneity of these diseases (80).

In addition to T- and B-lymphocytes, several other types of immune cells are involved in autoimmune disease development. Neutrophils kill extracellular bacteria by producing neutrophil extracellular traps (NETs), which are composed of DNA, histones, myeloperoxidase (MPO), and other components that play important roles in innate immune responses (81). However, recent evidence has suggested that neutrophils are linked to autoimmune disease pathogenesis because the substances present in NETs can serve as autoantigens and trigger autoimmune reactions; for instance, IL-8 production and exposure to citrullinated autoantigens was shown to exacerbate joint damage by enhancing the inflammatory response of RA synovial fibroblasts (82). Similarly, M1 macrophages in the synovium of patients with RA can release chemokines to promote the recruitment of more inflammatory cells and production of more inflammatory factors such as IL-1, TNF-α, and IL-6, leading to synovial and osteoarticular destruction (83). Dendritic cells (DCs) are important immune response conductors. Notably, heterogeneous DCs subpopulations and significant alterations in their function, such as defective migration and impaired phagocytosis in patients with SLE and RA, have been identified in various autoimmune diseases, partially explaining the pathological diversity of autoimmune diseases (84).

Given the diversity of immune cell phenotypes observed in autoimmune diseases, restoring the normal function of immune cells or eliminating dysregulated immune cells has been proposed as an effective strategy for treating autoimmune diseases. Currently, B-cell depletion therapy is emerging as a promising therapeutic approach, offering the potential to mitigate the side effects commonly associated with broad-spectrum immunosuppressants (85). Autoimmune diseases are highly heterogeneous and dynamic, resulting in various types of damage owing to the breakdown of immune tolerance and disruption of the immune system (86). Consequently, a comprehensive exploration of the abnormal alterations in the immune systems of patients with autoimmune diseases is imperative for developing more targeted therapeutic interventions in the future.

### 2.3 The mechanisms of immune tolerance

Immune tolerance refers to the phenomenon that immunologically active cells cannot be activated when stimulated by specific antigens, resulting in specific immune non-response (87), which is key to protecting the host tissues from destruction by its immune system without affecting the overall function of the adaptive immune response (88). Although contradicting, immune tolerance is essential for preventing immune cells from attacking the body tissues, thereby reducing the risk of autoimmune diseases. However, its persistence in chronic inflammation, tumors, and other pathological conditions can exacerbate disease progression and enable tumor cell survival (89).

Immune tolerance is categorized into central and peripheral according to the different periods of its formation, with the former being established during embryonic and postnatal development of the central immune organs (thymus and bone marrow) by eliminating autoreactive T- and B-lymphocytes through a complex mechanism of negative selection and clonal deletion. Defects in negative selection disrupt central tolerance, allowing the persistence of self-reactive lymphocytes that leads to autoimmunity in the body, thereby greatly increasing the probability of autoimmune disease (90). During the establishment of central tolerance, mTECs can express and present tissue-restricted self-antigens to T-cells through the expression of an autoimmune regulator (AIRE), a transcriptional activator that induces apoptosis following the high affinity binding of the T-cell receptor (TCR) to the self-antigen peptide-MHC molecular complex on the surface of mTECs (91). In addition, some autoreactive T-cells combine with self-antigens to develop natural Tregs (nTregs) with immunosuppressive effects; therefore, mTECs and their AIREs are fundamental and indispensable in the establishment of central tolerance (92). Negative selection also occurs during B-cell development when the functional B-cell receptor (BCR) complex is expressed on its surface for the first time; its high affinity binding to self-antigens leads to clone clearance and destruction of self-reactive B-cells (93).

The establishment of central tolerance can eliminate most self-reactive cells; however, some cells escape negative selection and clonal clearance, resulting in a significant number of cells that are unable to accurately distinguish nonself from self in healthy individuals, posing a potential risk of triggering autoimmune responses. Therefore, peripheral tolerance mechanisms are necessary for preventing autoimmune diseases (87). Peripheral tolerance refers to a state of immune nonresponsiveness or low response of mature T- and B-cells to endogenous or exogenous antigens through a variety of peripheral mechanisms (94). Clonal anergy is a common mechanism of peripheral tolerance, in which mature T- and B-cells with TCRs or BCRs on their surface that are capable of binding to antigens, are not adequately activated owing to the lack of costimulators or T-cell assistance, thus resulting in an unresponsive state in which they are prone to apoptosis (95). Another key mechanism of peripheral tolerance is the suppressive cell population, of which the most important are Tregs, which can be categorized into natural and peripherally induced Tregs, with the former exerting their immunosuppressive function through direct cell-to-cell contact, whereas the latter through the secretion of various cytokines such as IL-10 and TGF-β (96, 97).

## 3 The close link between epigenetics and autoimmune diseases

### 3.1 DNA modification and autoimmune diseases

Researchers have identified more than 10 different chemical modifications in DNA, each of which plays a unique role in biological development, aging, and disease (49). Of all the types of DNA modifications, methylation was the first to be discovered and the most intensively studied. DNA methylation can occur on adenine (A), guanine (G), and cytosine (C) (98). The DNA methyltransferase (DNMT) family acts as the “writer” of methylation modification, transferring methyl groups to the 5′-position of the cytosine, thus forming 5-methylcytosine (5mC) (99). DNMT3A and DNMT3B are *de novo* methyltransferases responsible for establishing DNA methylation, whereas DNMT1 is responsible for maintaining methylation after semi-conservative replication (100). DNA demethylation can be active or passive. The former refers to the progressive oxidation of 5mC by ten-eleven translocation (TET) cytosine dioxygenase, which is specifically recognized by the thymine DNA glycosylase that removes the oxidized product, followed by base excision repair to regenerate cytosine (101). The latter refers to the inability to maintain the methylated state of the new chain due to the inhibition of DNMT activity, resulting in the gradual dilution of 5mC in the genome (102). DNA methylation “readers” usually have a methyl-CpG binding domain (MBD); however, some transcription factors lacking MBDs were recently reported to also have the ability to bind to methylated DNA, thereby exerting downstream biological effects and regulating gene expression (50).

N6-methyldeoxyadenosine (6mA) was conventionally perceived as exclusively operational within prokaryotic organisms. Nevertheless, advances in detection methodologies with enhanced sensitivities have revealed the presence of 6mA in the genomic DNA of multicellular eukaryotic organisms. Their abundance and distribution in the genome exhibits significant disparities across diverse species (103). Similar to 5mC, the 6mA modification has its own “writer,” “eraser,” and “reader” (104). Methyltransferase like 4 (METTL4), which is a homolog of the *Caenorhabditis elegans* 6mA methyltransferase DAMT-1, is presumed to be the mammalian 6mA methyltransferase and has the ability to catalyze mitochondrial DNA (mtDNA) methylation (105). The human alkB homologue 1 (ALKBH1) and ALKBH4 proteins are recognized as 6mA demethylases, the mechanism of which involves the oxidation of the methyl group of 6mA with oxygen to regenerate unmodified adenine (106). Currently, investigations of human 6mA “readers” have primarily focused on mtDNA, with scant information available regarding the specific functions of 6mA in mammalian systems; however, given its diverse and significant role in prokaryotes, the exploration of the biological functions of 6mA in eukaryotes may emerge as a research hotspot in the field of epigenetics in the foreseeable future.

Decades of research has firmly established the correlation between DNA methylation and autoimmune diseases, with alterations in the DNA methylation spectrum observed across various diseases and associated with disease subtypes, activity levels, and patient responses to therapeutic interventions, thereby augmenting the potential of DNA methylation as a novel clinical biomarker (107). Interferon (IFN) is a cytokine involved in the regulation of the immune response. Patients with SLE exhibit significantly reduced methylation levels in their *IFN* genes, leading to increased IFN expression levels and exacerbation of nephritis and central nervous system symptoms (108). Patients with RA undergoing methotrexate treatment have unique DNA methylation profiles that determine their response to medication. Specifically, individuals exhibiting elevated levels of DNA methylation in whole-blood leukocytes tend to have increased disease activity and are more likely to develop resistance to methotrexate after 3 months of treatment (109).

Notably, some DNA-modifying enzymes have been found to play unexpected roles in other areas such as the posttranslational modification of proteins. Recent research has shown that METTL9 can catalyze histidine methylation, suggesting that DNA-modifying enzymes may play additional roles in autoimmune diseases by regulating intracellular biochemical reactions in unconventional ways, expanding our understanding of their potential function beyond traditional pathways (110). To further elucidate the pathophysiological role of DNA methylation in autoimmune diseases, large-scale epigenome-wide studies are needed, and an in-depth investigation is imperative for determining whether these modifications can serve as valuable epigenetic markers. This exploration has the potential to identify novel targets for the diagnosis and personalized treatment of patients with autoimmune diseases.

### 3.2 Histone modification and autoimmune diseases

**Figure 3:**
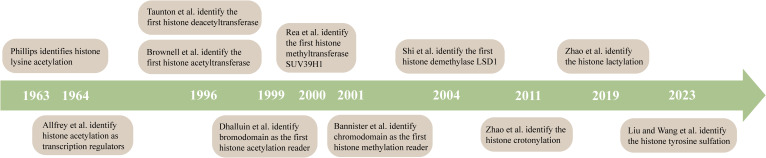
A timeline for studying histone modifications. Phillips identifies histone lysine acetylation (111). Allfrey et al. identify histone acetylation as transcription regulators (112). Taunton et al. identify the first histone deacetyltransferase (113). Brownell et al. identify the first histone acetyltransferase (114). Dhalluin et al. identify bromodomain as the first histone acetylation reader (115). Rea et al. identify the first histone methyltransferase SUV39H1 (116). Bannister et al. identify chromodomain as the first histone methylation reader (117). Shi et al. identify the first histone demethylase LSD1 (118). Zhao et al. identify the histone crotonylation (119). Zhao et al. identify the histone lactylation (120).

The posttranslational modification of histone amino acid residues is an extensively researched classical epigenetic mechanism (**Figure 3**). A wide range of chemical modifications exist, including methylation, acetylation, and phosphorylation (55) Notably, the newly discovered sumoylation, crotonylation, lactylation, and many other modifications (121) are all controlled by the corresponding “ writer” that adds the modification, “eraser” that removes the modification, and “reader” that recognizes the modification and enables the conduct of a specific biological function (122). Euchromatin, which is characterized by active gene expression, typically exhibits high levels of histone lysine acetylation. Regarding the underlying mechanism of action, acetylation neutralizes the positive charge carried by lysine residues, thereby reducing the affinity with negatively charged DNA. Consequently, a relaxed and open chromatin structure is formed, which facilitates the binding of transcription factors to DNA and promotes gene expression (123). Conversely, histone deacetylation creates a closed chromatin conformation and reduces transcriptional activity. Histone acetyltransferases (HATs) are “writers” of histone acetylation that can transfer the acetyl group on acetyl coenzyme A to lysine residues, and based on their sequence and structure, they are divided into three families, p300-CBP (CREB-binding protein), GNAT (Gcn5-related N-acetyltransferase), and MYST (MOZ, Ybf2/Sas3, Sas2, Tip60) (124). Histone deacetylases (HDACs) are the “erasers,” and are classified into four classes; among them, classes I, II, and IV are all Zn^2+^-dependent enzymes, whereas class III are nicotinamide adenine dinucleotide (NAD+)-dependent (125). The “readers” of histone acetylation contain domains that can accommodate specific acetylation modifications. After binding to histones, they can either recruit transcription factors to promote gene expression or serve as a barrier to prevent transcription factor binding (56). Another common modification, histone methylation, involves a complex mechanism that regulates gene transcription. Histone methylation can regulate transcriptional activity in both directions, depending on its levels and the specific amino acid sites (126).

Abnormal histone modifications play a critical role in the pathogenesis of various autoimmune diseases, thus establishing these modifications as clinical markers for the diagnosis and prognosis of diseases (127). TTNF-α-induced protein 3 (TNFAIP3) can inhibit inflammatory responses by negatively regulating the NF-κB inflammatory signaling pathway; however, the transcriptional activity marker trimethylated lysine 4 of histone H3 (H3K4me3) has been shown to be reduced in the *TNFAIP3* gene promoter of CD4+ T-cells in patients with SLE, resulting in a significant decrease in the *TNFAIP3* mRNA level and overexpression of IFN-γ and IL-17, thereby exacerbating the inflammatory response and promoting SLE pathogenesis (128). In addition, H3K4me3 and acetylated lysine 27 of histone 3 (H3K27ac) marks in IFN-responsive genes in the monocytes of patients with systemic sclerosis (SSc) were found to be increased to varying degrees, thus promoting the expression of these genes. In particular, an increase in myxoma resistance protein 1 (*MX1*) gene expression was reported to lead to severe lung function impairment and increased mortality in patients with SSc (129). Notably, histone modification is a reversible process, highlighting the potential of employing histone-modifying enzymes to restore abnormal histone modifications to their native state, and has emerged as a promising therapeutic strategy for addressing autoimmune diseases (130).

### 3.3 Chromatin remodeling and autoimmune diseases

The chromatin of eukaryotes not only carries genetic information but also contains a large amount of epigenetic information. The basic structural unit of chromatin, the nucleosome, comprises DNA wrapped around a core octamer consisting of two molecules, each of the histones H2A, H2B, H3, and H4 (131). Chromatin is found in two distinguishable forms: euchromatin, which is less condensed and has a high transcriptional activity, and heterochromatin, which is more compact and has a low transcriptional activity (132). Chromatin remodeling is a crucial epigenetic regulatory mechanism that modulates gene expression at the chromatin level by dynamically altering the open or closed state of chromatin, thus allowing or inhibiting the binding of transcription factors (133). Adenosine triphosphate (ATP)-dependent chromatin-remodeling complexes are major mediators of chromatin remodeling, dynamically regulating the chromatin structure by using energy generated from ATP hydrolysis to move, remove, or replace nucleosomes (134). The structure of ATP-dependent chromatin remodeling complex is highly conserved in eukaryotes, and these complexes can be divided into four families according to the structural characteristics of the core ATPase subunit: switching defective/sucrose nonfermenting (SWI/SNF), imitation switch (ISWI), inositol requiring 80 (INO80), and chromodomain helicase DNA-binding (CHD), all of which play unique roles in regulating transcriptional activity (135). For example, the SWI/SNF complex comprises various structural domains capable of interacting with DNA or histones, regulating the accessibility of transcription factors to chromatin by mediating the ejection, sliding, and removal of nucleosomes on DNA, thus promoting or repressing gene expression (136). Furthermore, these remodeling complexes can coordinate with histone modifications and variants to regulate chromatin remodeling, thus finely regulating biological processes such as DNA replication, repair, and transcription (137).

A close relationship between chromatin-remodeling complexes and autoimmune diseases has also been discovered. Studies have shown that small-molecule inhibitors targeting SWI/SNF can attenuate T-cell depletion by altering transcription factor binding motif sites; therefore, inhibiting SWI/SNF activity may drive the progression of autoimmune disease by affecting the activation and exhaustion of T-cells (138). T helper 17 (Th17) cells participate in inflammatory responses by producing IL-17 and IL-21 and recruiting inflammatory cells, which play important roles in many autoimmune diseases such as psoriasis and multiple sclerosis (MS) (139). SRG3 is a scaffold protein that has been shown to control the stability of the SWI/SNF complex in mice. The secretion of IL-17A and IL-17F by CD4+ T-cells is greatly reduced in mice lacking SRG3, thereby affecting the generation and differentiation of Th17 cells (140). The chromatin remodeler polybromo-1 (PBRM1) belongs to the SWI/SNF family, and some patients with Crohn’s disease and ulcerative colitis (UC) have been found to have low levels of PBRM1 expression, which leads to an increase in the expression of IFN and several proinflammatory cytokines through the activation of the retinoic acid-inducible gene-I-like receptor (RLRs) signaling pathway, thus exacerbating inflammation in patients (141). Given the limited research on the relationship between ATP-dependent chromatin-remodeling complexes and autoimmune diseases, further investigations in this domain are warranted to provide new insights into the pathophysiological mechanisms underlying autoimmune diseases (142).

### 3.4 RNA modification and autoimmune diseases

With the rapid development of detection technologies, more than 100 modifications have been identified in different types of RNA. As critical components of epigenetics, these chemical modifications significantly influence the regulation of gene expression at the posttranscriptional level by affecting the stability, localization, and splicing of RNA (51). Among all RNA species, transfer RNA (tRNA) is known to exhibit the highest degree of modifications and most abundant modification types, including N1-methyladenosine (m1A), pseudouridine (Ψ), and N7-methylguanosine (m7G), which are crucial to its various biological functions (52). Notably, m6A is among the known eukaryotic mRNA modifications. Whole-transcriptome localization analysis revealed that the distribution of m6A is relatively conserved and sequence-specific, and mainly enriched in the 3′ untranslated region (3′ UTR) and near the stop codon (143). The “writer” responsible for depositing the m6A modification is the multi-subunit methyltransferase complex, which consists of the key catalytic subunit METTL3, RNA-binding facilitator METTL14, and many other accessory subunits (144). The m6A modification is a dynamic and reversible process, and the active demethylation is mainly catalyzed by the “erasers”, fat-mass, and obesity-associated protein (FTO) (145) and ALKBH5 (146), which are located in the nucleus and affect mRNA metabolism. RNA-binding proteins, including YT521-B homology (YTH) domain family 1-3 (YTHDF1-3), and YTH domain-containing protein 1-2 (YTHDC1-2) are “readers” (147) that selectively recognize and bind m6A modifications in target mRNAs, thereby affecting the biological function of cells (148). YTHDF1 promotes the translation efficiency of mRNAs containing m6A modifications by interacting with the translation initiation factor eIF3 (149). In contrast, YTHDF2 binds to m6A modification in 3’UTR to promote mRNA degradation (150), while YTHDF3 can interact with YTHDF1 and YTHDF2 to facilitate their respective functions (151). Further RNA modification enzymes are expected to be identified and studied in the future, enabling the exploration of their potential functions.

These results demonstrated that m6A can regulate gene expression post-transcriptionally by influencing RNA metabolism, thereby participating in various biological processes and playing a unique role in human autoimmune diseases (152). Studies have revealed a significant decrease in *ALKBH5* and *FTO* mRNA expression, whereas a marked increase in that of METTL3, the enzyme that activates the NF-κB signaling pathway, thus exacerbating the inflammatory response in patients with RA (153). Furthermore, m6A regulates the differentiation of naïve T-cells and influences their function. Mice with *METTL3*-knockout Tregs developed severe autoimmune disease after weaning, indicating that Tregs lose their immunosuppressive function due to the lack of the m6A modification (154). Given the essential function of m6A in various immune cells and autoimmune diseases, the relationship between other RNA modifications and autoimmune disease pathogenesis warrants further investigation in the future. These modifications could serve as new diagnostic markers of disease and facilitate the development of innovative approaches for the treatment of autoimmune diseases.

### 3.5 The impact of epigenetics on immune tolerance

The establishment and maintenance of immune tolerance require the precise regulation of multiple mechanisms that coordinately control the elimination or inactivation of self-reactive cells, thereby creating a robust barrier against pathological autoimmune responses. However, owing to the complexity of these mechanisms and heterogeneity of immune-related diseases, a large number of questions remain unanswered (155, 156). Extensive epigenetic studies have demonstrated that epigenetic regulation is indispensable for the establishment and maintenance of immune tolerance. For example, during the establishment of central immune tolerance, specific epigenetic modifications in AIRE are essential for regulating the active transcription of peripheral tissue genes (PTGs) in mTECs (92). Heinlein et al. demonstrated that the histone acetyltransferase KAT7 is a key protein for the AIRE-mediated transcription of PTGs in mTECs and induction of immune tolerance. The levels of H3K14ac, which is a marker of transcriptional activation, in the mTEC genome of KAT7-knockout mice were significantly decreased (157). Although the expression of AIRE itself was not affected, the accessibility of chromatin around the promoter of its target gene was reduced, leading to the downregulation of gene expression. During early development, Tregs with strong immunosuppressive functions form super enhancers (SEs), which are cis-regulatory elements with excellent transcriptional activation properties, characterized by highly dense transcriptional activity markers H3K27ac and H3K4me1, which play an essential role in promoting the expression of Treg marker genes (158).

These results indicate that epigenetic modifications can alter the levels of intracellular gene expression and transcription products that control the development, differentiation, and function of immune cells, thereby disrupting immune tolerance and aberrant clearance of self-reactive lymphocytes, causing pathological autoimmune responses, and ultimately driving the development of autoimmune diseases (159, 160). Enhancer of zeste homolog 2 (EZH2) is a histone methylation “writer” that can catalyze H3K27me3, a transcriptional repression marker, to inhibit the expression of target genes, thus playing a crucial role in immune tolerance and homeostasis (161). Researchers have found that CD4+ T-cells from patients with RA have low levels of EZH2, which downregulates the expression of RUNX1, a key transcription factor for Tregs, ultimately inhibiting Treg differentiation, disrupting peripheral immune tolerance, and exacerbating the inflammatory response in the joints of patients with RA (162). Hence, abnormal changes in the content or function of epigenetically modified enzymes can destroy immune tolerance by affecting the function of immune cells, thereby mediating a variety of autoimmune diseases. In addition, abnormal epigenetic modifications can affect central tolerance mechanisms and trigger autoimmune diseases. Studies have shown that mTEC development requires the histone deacetylase sirtuin 6, lack of which impairs DNA replication and proliferation in mTECs. Concurrently, sirtuin 6 loss results in abnormal expression of PTGs, inhibited thymocyte and nTreg development, causing thymus atrophy and destruction of central immune tolerance, and eventually inducing autoimmune diseases, which manifest as multiorgan lymphocyte infiltration and high autoantibody titers (163). Notably, these epigenetic modification enzymes may not alter the epigenetic modifications of the genome but may affect cell development and function through additional mechanisms, thus driving the occurrence and development of autoimmune diseases (164, 165). Future research should carefully explore these enzymes and understand their precise role in the regulation of cells and molecules, which may offer valuable insights into the development of novel treatments for autoimmune diseases.

### 3.6 Targeting epigenetic modifications of immune molecules

**Figure 4:**
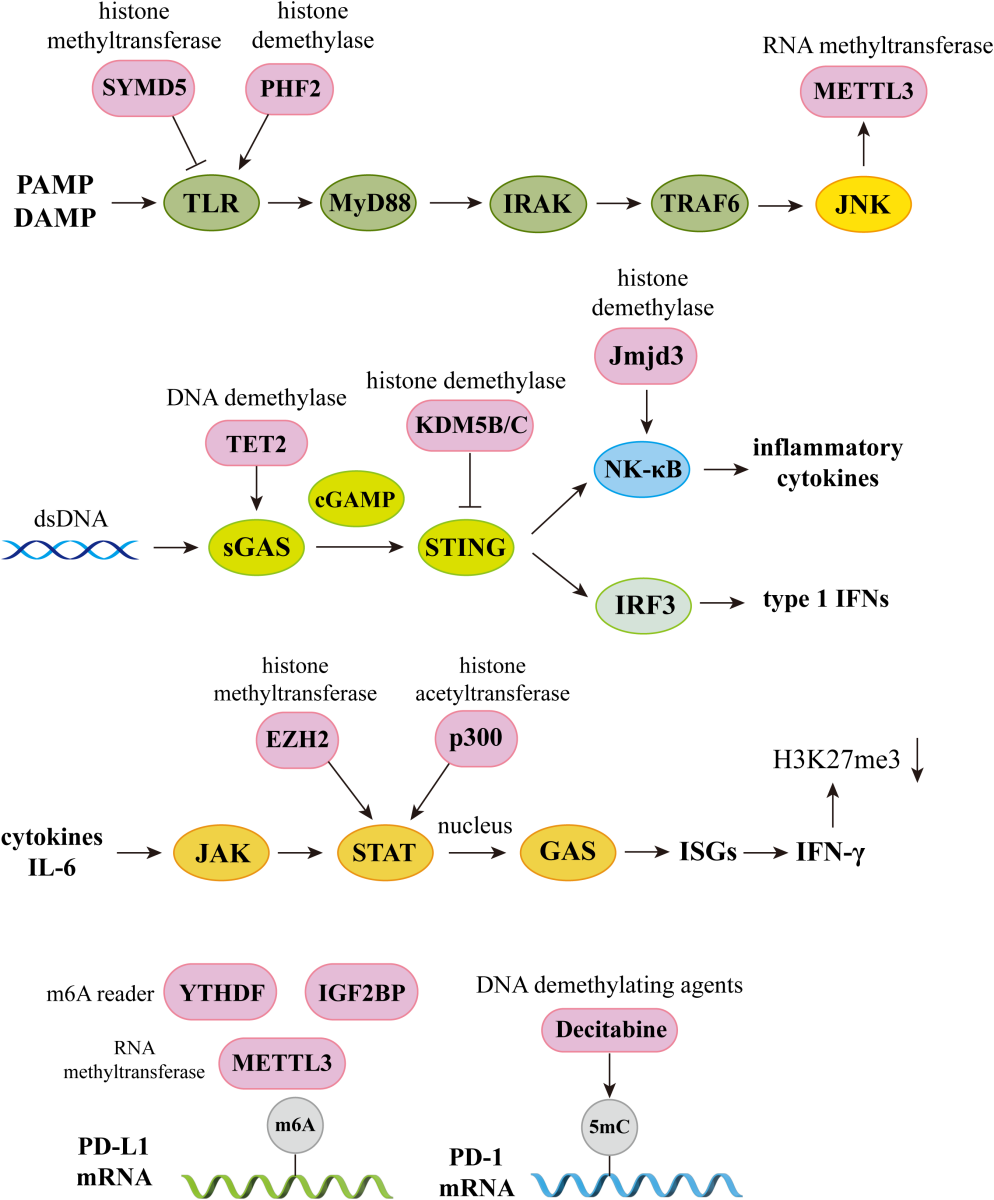
The crossroads of epigenetics and immune pathways. Epigenetics plays an integral role in the regulation of immune pathways. In the TLR signaling pathway, histone methyltransferase SYMD5 catalyzes H4K20me3 to repress TLR4 expression; histone demethylase PHF2 activates TLR4 expression by removal of H3K9me1. Knockdown of JNK will inhibit m6A and METTL3 expression; In the sGAS-STING signaling pathway, DNA demethylases TET2 elevate cGAS levels, histone demethylases KDM5B and KDM5C inhibit STING expression. Histone demethylase Jmjd3 upregulates NF-kB-mediated inflammatory cytokine levels by removal of H3K27me3; In the JAK-STAT signaling pathway, activator of transcription STAT recruits histone acetyltransferases and chromatin-remodeling enzymes, and histone methyltransferase EZH2 and histone acetyltransferase p300 elevate STAT levels. IFN-γ produced by this signaling pathway can induce H3K27me3, which is associated with gene repression; In the PD-1 and PD-L1 pathway, knockdown of METTL3 abolishes m6A modification and reduces stabilization of PD-L1 mRNA, and m6A reader IGF2BP and YTHDF regulates RNA stability and expression levels of PD-L1. DNA methyltransferase inhibitor 5-aza-2’ deoxycytidine (decitabine) enhances PD-1 expression. PAMP, pathogen associated molecular pattern; DAMP, damage associated molecular pattern; TLR, Toll-like receptors; IRAK, interleukin-1 receptor-associated kinase; TRAF6, Tumor necrosis factor receptor-associated factor 6; IRF3, Interferon regulatory factor 3; ISGs, Interferon-stimulated genes.

With the progress of research, the immune system has emerged as an excellent model for analyzing the regulatory mechanisms of epigenetics. The close connection between immune cell function and epigenetics undoubtedly makes this crosscutting area promising for exploration and development. The regulation of immune molecules by epigenetic modifications can elucidates these interactions in the subcellular level. The crossroads between epigenetic and immunopathways are important in inflammatory regulation, disease development, and therapeutic strategies (**Figure 4**).

The cyclic GMP-AMP synthase (cGAS)-stimulator of interferon genes (STING) signaling pathway regulates the immune response by promoting the secretion of type I IFN (166). Several studies have shown that chronic and sustained aberrant activation of this pathway is closely related to autoimmune disease pathogenesis (167). Clinical studies have revealed that activated peripheral blood mononuclear cells (PBMCs) of patients with SLE have a higher expression of cGAS, leading to increased serum levels of type I IFN and mediating the inflammatory response and damage to the body (168). This pathway is regulated through various epigenetic mechanisms, with high methylation levels detected in the promoter region of cGAS and STING in many different types of tumor cells. STING expression is inhibited by the histone H3K4 lysine demethylases KDM5B and KDM5C. This epigenetic silencing mechanism prevents the activation of the pathway and inhibits the immune activity of antitumor T-cells, allowing cancer cells to escape immune surveillance (169). In addition, DNA demethylase, TET2, modulates anti-tumor immunity by elevating cGAS levels in tumor cells (170).

Programmed death 1 (PD-1) and programmed death ligand 1 (PD-L1) play critical roles in regulating the immune response and T-cell activation state, and modulate central and peripheral tolerance by transducing immunosuppressive signals (171). Abnormal expression of these genes may lead to the abnormal activation of autoimmune T-cells and the development of several autoimmune diseases (172). Downregulation of PD-1 on Tregs in SLE patients results in reduced cell numbers and impaired immunosuppression (173). Moreover, synovial T-cells and macrophages in patients with RA were demonstrated to have significantly higher levels of PD-1/PD-L1 expression than those in healthy controls, with proinflammatory cytokines such as IFN-γ and TNF-α contributing to the upregulation of these costimulatory molecules to some extent (174). Studies have shown that PD-L1 expression is regulated by specific epigenetic enzymes, such as METTL3, the “writer” of m6A, which increases PD-L1 mRNA expression, promoting bladder cancer immune escape (175).

In addition, despite the regulation of a large number of immune molecules by epigenetic modifications, the complex mechanisms governing these interactions remain unclear. Given the critical role of epigenetic modifications in the regulation of numerous signaling pathways, studies delineating the effect of these modifications on immune regulation may open new avenues for improved immunotherapy.

## 4 Epigenetic biomarkers and therapeutic approaches in autoimmune diseases

### 4.1 Epigenetic biomarkers in autoimmune diseases

Epigenetics is increasingly recognized as a crucial factor in autoimmune disease pathogenesis, and researchers have identified a variety of epigenetic modifications as promising candidates for autoimmune disease biomarkers, which could help guide clinical diagnosis, treatment, and prognosis (176). Decades of research has demonstrated that compared with healthy individuals, patients with autoimmune diseases exhibit significant epigenetic changes in their genomes, which are strongly associated with disease phenotype and activity. Detecting changes in DNA methylation or histone modification is possible during the early stages of certain diseases, thus providing a basis for the early prediction, diagnosis, and prognosis of diseases (**Table 3**) (202).

**Table 3:** Epigenetics as a biomarker for autoimmune diseases.

Epigenetics	Disease	Biomarkers	Clinical significance	Reference
DNA methylation	Systemic lupus erythematosus (SLE)	Hypomethylation within CD40-ligand promotor and enhancer regions	hypomethylation is associated with disease activity in female SLE patients	(177)
		Impaired DNA methylation in CD4+T and B cells	The percentage of 5mC inversely correlates with the disease activity of lupus patients	(178, 179)
		hypermethylation at Interferon-induced protein 44-like (IFI44L) gene in CD4+T cells	Overexpression of IFI44L can amplify IFN signaling and enhance the immune response	(180)
		The level of DNMT1 mRNA expression in CD4+ T cells from SLE patients were significantly decreased	UVB enhanced DNA hypomethylation via inhibiting DNMT1 catalytic activity, causing the patient’s condition to deteriorate	(181)
	Rheumatoid Arthritis (RA)	T cells exhibit overall DNA hypomethylation; naïve T cells exhibit increased methylation levels, which diminish during differentiation into effector/memory T cells	Altered DNA methylation may facilitate the accelerated degradation and premature senescence of naïve and memory CD4+ T cells, causing abnormal T cell activation and senescence	(182, 183)
		Hypermethylation at CD1C and hypomethylation at TNFSF10 genes	TNFSF10 overexpression causes bone and joint damage	(184)
		Decreased levels of DNMT1 in synovial fibroblasts (SFs), leading to an overall decrease in DNA methylation	SFs became an aggressive phenotype, producing pro-inflammatory cytokines and making the disease worse	(185)
	Primary Sjögren’s syndrome (pSS)	Significant hypomethylation of interferon (IFN)-regulated genes in whole blood and CD19+ B cells	Hypomethylation of IFN-regulated genes increased their expression, activating the IFN signaling pathway and mediating autoimmune responses	(186)
		Reduced DNA methylation levels in minor salivary gland (MSG) epithelial cells	Hypomethylation are associated with lymphocyte infiltration or anti-SSB/La antibody production	(187)
	Systemic sclerosis (SSc)	CD4+ T cells in female SSc patients exhibit hypomethylation and overexpression of CD40L	The interaction of CD40L with CD40 stimulates B cells and fibroblasts, promoting autoantibody production and tissue fibrosis	(188)
		The methylation levels of the CD11a regulatory sequences were lower, leading to increased expression levels in CD4+ T cells	CD11a levels were found to be positively correlated with disease activity, stimulating B cells to produce IgG	(189)
Histone modification	SLE	Global histone H3 and H4 hypoacetylation in active lupus CD4+ T cells	the level of H3 acetylation is negatively correlated with the disease activity	(190)
		Histone methylase EZH2 and H3K27me3 levels were increased in CD4+ T cells in lupus	Overexpression of EZH2 resulted in increased CD4+ T cell adhesion	(191)
		H3K27me3 levels in the Transcription factor B cell lymphoma 6 (BCL6) promoter region were decreased	Decreased H3K27me3 levels promoting regulator of T follicular helper (Tfh) cells differentiation	(192)
	SSc	Global histone H4 hyperacetylation and global histone H3K9 hypomethylation B cells	Global H4 acetylation was positively correlated with disease activity	(193)
RNA methylation	SLE	mRNA levels of METTL3, FTO, ALKBH5, and YTHDF2 in peripheral blood from SLE patients were significantly decreased	The levels of ALKBH5 mRNA were associated with anti-dsDNA, antinucleosome, rash, and ulceration	(194)
		mRNA expression of MTEEL14, ALKBH5 and YTHDF2 was reduced in SLE patients	Decreased YTHDF2 was associated with disease activity	(195)
		m5C levels were decreased in CD4+ T cells of patients with SLE	Lower m5C levels are associated with severe disease activity	(196)
		RNA methylase NSUN2 was markedly decreased in CD4 + T cells from SLE patients	NSUN2 catalyzes the modification of IL-17A mRNA, which is significantly implicated in the pathogenesis of SLE	(197)
	RA	The levels of METTL14 and m6A are decreased in the PBMCs of patients with active RA	m6A are negatively correlated with the DAS28 score and IL-6 and IL-17 levels	(198)
		the expression of METTL3 in macrophages was significantly increased	METTL3 level was positively correlated with the RA activity marker	(199)
	pSS	The mRNA levels of m6A writers (METTL3 and RBM15), erasers (ALKBH5 and FTO), and readers (YTHDF1, YTHDF2, YTHDF3, YTHDC1, and YTHDC2) were all significantly higher in PBMCs from pSS patients	The mRNA level of YTHDF1 in PBMCs was negatively correlated with the EULAR Sjögren’s syndrome disease activity index (ESSDAI) score. Increased mRNA level of ALKBH5 in PBMCs was a risk factor for pSS	(200)
	Ankylosing spondylitis (AS)	m6A levels and expression of METTL14, WTAP and ALKBH5 were significantly reduced in T cells	Reduced METTL14 levels inhibited the transcription of autophagy-related genes, increasing levels of IL-17A, IL-23, and TNF-α	(201)

The DNA methylation profile of various cells is significantly altered in patients with SLE; for example, CD4+ T-cells and B-cells usually exhibit global hypomethylation, particularly in immune-related genes. Therefore, the DNA methylation of specific genes may serve as a potential biomarker for SLE (178). In addition, interferon-induced protein 44-like (IFI44L), an interferon-regulated gene that can amplify IFN signaling and enhance the immune response is hypomethylated in CD4+ T-cells from patients with SLE; thus, the DNA methylation of the IFI44L promoter can serve as a potential blood biomarker for diagnosing SLE with high sensitivity and specificity (180). In addition to immune cells, DNA methylation alterations can occur in cells with typical significance in autoimmune diseases. For instance, the decreased levels of DNMT1 in synovial fibroblasts (SFs) from patients with RA can lead to an overall decrease in DNA methylation, which can induce an aggressive SF phenotype that produces proinflammatory cytokines, such as IL-6 and TNF, thereby promoting the development of RA (185). Similarly, reduced DNA methylation levels were observed in minor salivary gland (MSG) epithelial cells of patients with Sjögren’s syndrome (SS), and these changes were associated with lymphocyte infiltration and anti-SSB/La antibody production (187). To facilitate the detection of epigenetic modification changes in the early stages of an autoimmune disease or even before its onset, developing more efficient detection methods is required.

Abnormal alterations in histone modifications have been observed in the cells of patients with autoimmune diseases, and given these modifications are strongly associated with the onset and progression of the disease, they may serve as potential biomarkers for assessing disease risk and aiding in diagnosis (62). Studies have shown that the abnormal expression of several histone acetylases and deacetylases leads to the overall hypoacetylation of H3 and H4 in CD4+ T-cells from patients with SLE, with a negative correlation observed between the degree of H3 acetylation and disease activity (190). However, in patients with active lupus, hyperacetylation has been detected at specific genes, such as markedly elevated levels of H3 acetylation and an increase in H3K4me2 at the *CD70* gene, both of which promote its transcriptional activation, strengthening the immune response by promoting the activation and proliferation of T- and B-cells (203). In addition, H3K9 hypomethylation was detected in the B-cells of patients with SSc and CD4+ T-cells from patients with SLE; however, patients with SSc exhibited global histone H4 hyperacetylation, the level of which was positively linked to disease activity (193). Hence, epigenetic modifications appear to be as heterogeneous as autoimmune diseases, different diseases and cell types characterized by different types of modifications; therefore, the relationship between epigenetic modifications and autoimmune diseases requires further detailed exploration.

The importance of RNA modifications in the pathogenesis of autoimmune diseases has been gradually elucidated with the development of advanced sequencing technologies (152). Following the pharmacological inhibition of METTL3 in lupus model mice, the m6A modification on the transcription factor *Foxp3* mRNA was reduced, in turn facilitating RNA degradation, and resulting in impaired differentiation and immunosuppressive function of Treg cells as FOXP3 is indispensable for the development and function of Treg cells, thus exacerbating kidney injury in mice (204). Moreover, the expression of METTL3 in the macrophages of patients with RA was significantly increased, and was positively correlated with RA activity markers, such as C-reactive protein (CRP), suggesting the correlation of m6A with RA disease activity to some extent (199). These results showed that m6A and its modifying enzymes play crucial roles in immune cell differentiation and development and have the potential to drive autoimmune diseases, thus supporting their potential as biomarkers and therapeutic targets for managing this disease. Although research on RNA modifications is being rapidly conducted, developing more efficient and accurate sequencing technologies for comprehensively exploring other types of RNA modifications and their modifying enzymes is urgently needed to provide strong evidence for the development of new therapies.

### 4.2 Epigenetic therapeutic approaches in autoimmune diseases

With the in-depth study of epigenetic mechanisms, a new understanding of abnormal epigenetic changes in autoimmune diseases has been obtained, fueling an array of studies aimed at identifying clinical markers for early disease diagnosis and prognosis. Accordingly, the reversibility of epigenetic modifications, which holds great therapeutic potential, has led to the emergence of epigenetic therapy as a novel therapeutic approach for autoimmune diseases and the development of new drugs that may provide great benefits to patients (**Table 4**) (228).

**Table 4:** Epidrugs used in autoimmune disease.

Drug		Disease	Cell type	Effects	Improvement of symptoms	References
HDAC inhibitor	Trichostatin A	RA	PBMC	Inhibited production of TNF, IL-6 and IFN-γ		(205)
		SLE	T cell	Downregulated CD154 (CD40L) and IL-10, upregulated IFN-γ		(206)
		SLE	MRL-lpr/lpr mice splenocytes	Downregulated mRNA and protein levels of IL-12, IL-6, IL-10 and IFN-γ	Reduced proteinuria, glomerulonephritis, and spleen weight	(207)
		SLE	NZB/W mice T cell	Decreased production of IL-6 and increased TGF-β1 and Foxp3; decrease expression of renal MCP-1, MMP-9, and IL-6 mRNA	Decreased anti-dsDNA autoantibodies, protein excretion, renal IgG and C3 deposition, and pathologic glomerular disease	(208)
		T1D	T cell	Upregulated IFN-γ and its transcription factor Tbx21	Reduced the incidence of diabetes	(209)
		SSc	Fibroblast	Increased FLI1 and reduced type I collagen		(210)
		SSc	Fibroblast	Reduced production of type I and type III collagen		(211)
	MI192 (selective HDAC3 inhibitor)	RA	PBMC	Inhibited the production of TNF at high concentrations, and inhibited IL-6 dose-dependently		(205)
	ACY-738 (selective HDAC6 inhibitor)	SLE	T cell, B cell	Decreased expression of glomerular IL-10, TGF-β, and IL-6 mRNA; reduced levels of serum anti-dsDNA autoantibodies and IgG isotype	Inhibited immune complex-mediated glomerulonephritis and decreased SLE renal pathology	(212)
	Givinostat (ITF2357)	RA	FLS, macrophage	Suppressed FLS IL-6 production and reduced the stability of IL-6 mRNA in FLS and macrophages		(213)
		T1D	NOD mice T cell, DC, pancreatic β cells	Increased Treg cell subsets and their transcription factors Gata3 and FoxP3; decreased inflammatory DC subsets and their cytokines IL-6, IL-12, and TNF-α	Reduced diabetes incidence, increased insulin content	(214)
		SLE	NZB/W mice T cell	Reduced levels of serum anti-dsDNA autoantibodies and IgG isotype; decreased glomerular IL-6 mRNA dose-dependently	Decreased renal disease and glomerular immune complex deposition; increased the number of regulatory T cells and inhibited Th17 differentiation	(215)
	Vorinostat (SAHA)	MS	CD14+ monocyte-derived DC	Reduced CD80 and CD86, the maturation marker CD83, and HLA-DR; inhibited production of TNF-α, IFN-γ, IL-12p35, IL-6 and IL-23p40	Reduced CNS inflammation and demyelination	(216)
		RA	CIA mice T cell	reduced expression of NR1D1, IL-17 mRNA and protein	Decreased Th17 cell counts	(217)
		RA	FLS	Increased intracellular ROS levels, and expression and activity of caspase-3; reduced anti-apoptotic Bcl-2 proteins (Bcl-xL and Mcl-1), and phosphorylated IκBα and NF-κB p65	Increased FLS apoptosis	(218)
		T1D	NOD mice T cell, pancreatic β cells	Reduced levels of IL-1β and IFN-γ-induced proinflammatory, and cytokine-induced ERK phosphorylation	Reduced diabetes incidence, increased insulin content	(214)
		SLE	MRL-lpr/lpr mice splenocytes	Downregulated mRNA and protein levels of IL-12, IL-6, IL-10 and IFN-γ	Reduced proteinuria, glomerulonephritis, and spleen weight	(207)
		SLE	PBMC	Ameliorated DNA repair efficiency and decreased apoptosis		(219)
		SLE	MRL/lpr mice mesangial cell	Inhibited expression of TNF-α, IL-6, NO, and inducible NO synthase	Decreased spleen size and CD4-CD8- (double-negative) T cells; inhibited proteinuria and pathologic renal disease	(220)
		SLE	anti-CD3 activated T cell	Augmented apoptosis at high concentrations, inhibited proliferation of anti-CD3 activated T cells and reduced IL-6, IFN-γ, TNF-α and IL-2 at low concentrations	Ameliorated cytokine storm syndrome and avoided GVHD; combination of SAHA and anti-CD3 cured severe lupus glomerulonephritis	(221)
	THS-78-5	T1D	INS cell	Prevented IL‐1β‐mediated iNOS expression and NO release	Prevented IL-1β-induced metabolic dysfunction in pancreatic β cells	(222)
DNMT inhibitor	5’-aza-2’-deoxycytidine	MS	Treg cell	Elevated Foxp3 expression *in vivo* and *in vitro*; decreased proinflammatory cytokines	Ameliorated CNS inflammatory responses	(223)
		SSc	Fibroblast	Increased FLI1 and reduced type I collagen		(210)
		SSc	Fibroblast	Reactivated of DKK1 and SFRP1 transcription and reduced Wnt signaling		(224)
		RA	FLS	Upregulated expression of PTEN, decreased chemokines CCL-2, CCL-3, CCL-8, and proinflammatory cytokines IL-1β, IL-6, IL-17A	Decreased inflammatory cell infiltration into the synovium and reduced in paw swelling	(225)
	5’-azacytidine	RA	Mice B cell	Repressed Aicda gene expression, reduced CSR and impaired GC formation	Inhibited production of IgG1 antibodies and attenuated joint destruction	(226)
		SLE	MRL/lpr mice CD4 and CD8 T cell	DNA demethylation in CD4+T cells enhanced Foxp3 expression, and increased CD8+T cells cytotoxic activity	Ameliorated facial rash and skin lesions, reduced proteinuria	(227)

HDAC, histone deacetylase; PBMC, peripheral blood mononuclear cells; DNMT, DNA methyltransferase; SAHA, suberoylanilide hydroxamic acid; CD, cluster designation; DC, dendritic cells; IL, interleukin; TNF, tumor necrosis factor; FLS, fibroblast-like synoviocytes; NOD, nonobese diabetic; IFN-γ, interferon-γ; dsDNA, double stranded DNA; GVHD, graft versus host disease; EAE, experimental autoimmune encephalomyelitis; NF-κB, nuclear factor kappa B; CIA, collagen-induced arthritis; NR1D1, nuclear receptor subfamily 1 group D member 1.

HDAC inhibitors exhibit strong immunomodulatory functions in immune cells and have been widely studied as an epigenetic therapy for autoimmune diseases. One month after lupus model mice were treated with a selective HDAC6 inhibitor, lupus nephritis (LN) symptoms were significantly alleviated, and the deposition of IgG and C3 in glomeruli was significantly decreased. Furthermore, HDAC6 inhibitors have been shown to suppress various signaling pathways associated with B-cell activation and differentiation, thereby inhibiting the formation of plasma cells and germinal center (GC) response in lupus model mice (176). Another study demonstrated that belinostat, a pan-HDAC inhibitor, can suppress neuroinflammation by inhibiting M1 microglial activation and proinflammatory cytokine expression, thereby improving symptoms in experimental autoimmune encephalomyelitis (EAE) mice, thus indicating belinostat as a potential candidate for multiple sclerosis (MS) treatment (229).

In another example, the DNA methyltransferase inhibitor 5’-azacytidine (5’-azaC) has shown better efficacy in animal models of certain autoimmune diseases. 5’-azaC can mediate the demethylation of the *Ahr* gene, leading to the repression of *Aicda* gene expression, which is required for GC formation. Ultimately, mice with proteoglycan-induced arthritis were shown to produce fewer IgG1 antibodies, resulting in improved autoimmune arthritis (226). Targeted delivery of 5’-azaC to CD4+ or CD8+ T-cells in MRL/lpr mice resulted in lower levels of multiple inflammatory factors and significant amelioration of autoimmunity. DNA demethylation in CD4+ T-cells was suggested to increase Foxp3 expression and induce immunosuppressive properties in these cells (227). Peripheral application of the DNA methyltransferase inhibitor 5’-aza-2’-deoxycytidine to EAE mice suppressed central nervous system inflammation and reduced IFN-γ and IL-17 levels by increasing the number of Treg (223). However, owing to the high heterogeneity of autoimmune diseases and complexity of epigenetic mechanisms involved, some studies have shown that the DNMT inhibitor 5’-aza-2’-deoxycytidine can promote the expression of Foxp3 in Treg cells to a certain extent, thus strengthening its immunosuppressive function; however, the continuous use of inhibitors can lead to a decrease in DNMT1 activity, which will instead disrupt the function of Tregs and induce fatal autoimmunity in mice (230).

In conclusion, epigenetic therapies are an emerging and promising therapeutic approach; however, many limitations and challenges exist regarding the application of these therapies for treating autoimmune diseases; hence they remain largely in an exploratory phase. Numerous clinical trials evaluating epigenetic drugs have been conducted in the field of oncology. However, trials targeting autoimmune diseases remain sparse (**Table 5**). Further in-depth research on epigenetic mechanisms is warranted to develop highly specific targeted epigenetic drugs. The combination of epigenetic therapy and traditional immunotherapy will provide valuable insights into the treatment of autoimmune diseases.

**Table 5:** Summary of clinical trials of epigenetic modulators and immunotherapeutic agents.

Epigenetics	Mechanism	Agents	Chemical structures	Immune-therapeutic agent	Disease type	Trail
Histone acetylation	HDAC inhibitor	Domatinostat (4SC-202)	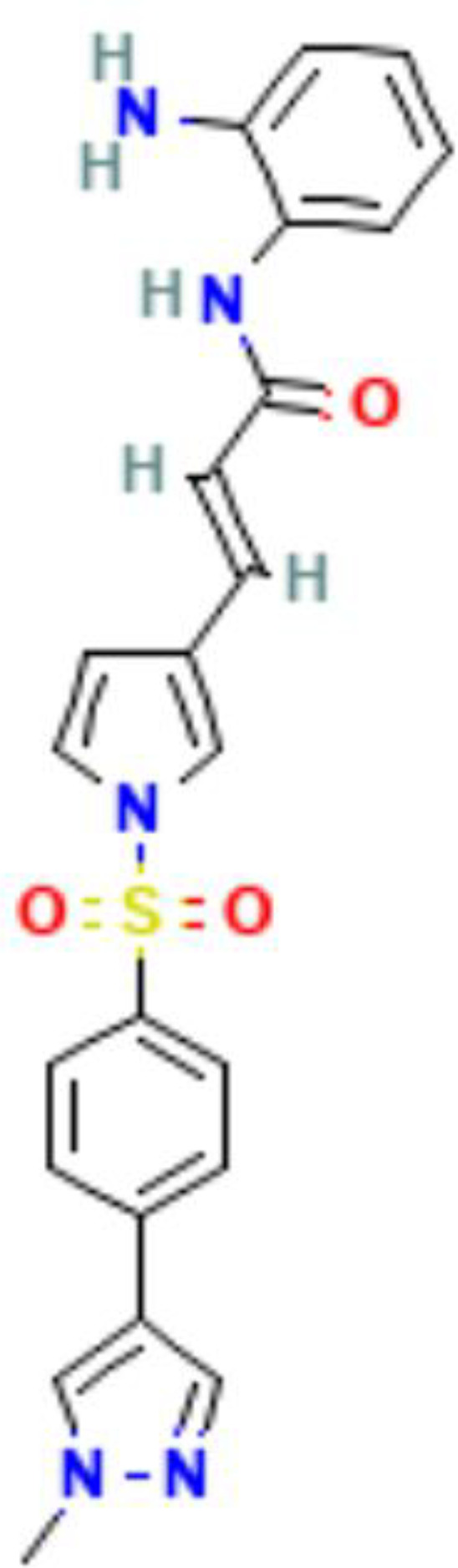	Avelumab	GastrointEstinal Cancers	NCT03812796
					Advanced Merkel Cell Carcinoma	NCT04393753
					Metastatic Merkel Cell Carcinoma	NCT04874831
				Nivolumab + Ipilimumab	Primary Melanoma	NCT04133948
		Entinostat	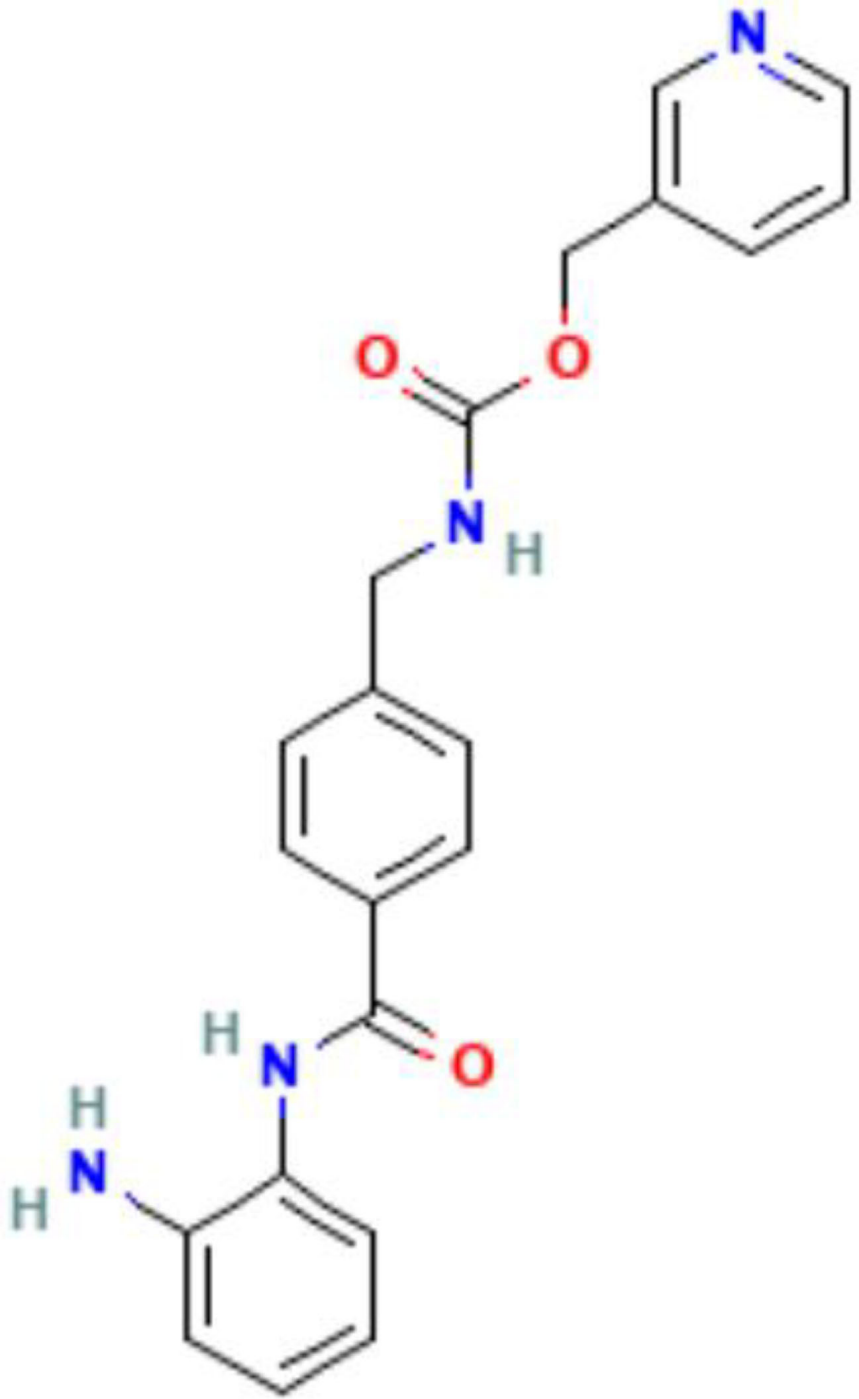		Advanced Prostate Cancer	NCT03829930
					Breast Cancer	NCT02820961
				Ipilimumab/ Nivolumab	HER2-Negative Breast Cancer	NCT02453620
				Erlotinib	Breast Cancer or NSCLC	NCT01594398
				Nivolumab	Colorectal Cancer	NCT03993626
				Nivolumab	Pancreatic	NCT03250273
				Avelumab	Advanced Epithelial Ovarian Cancer	NCT02915523
				Aldesleukin	Metastatic Kidney Cancer	NCT01038778
				Pembrolizumab	Bladder Cancer	NCT03978624
					Lymphoma	NCT03179930
					Melanoma	NCT03765229
		Vorinostat	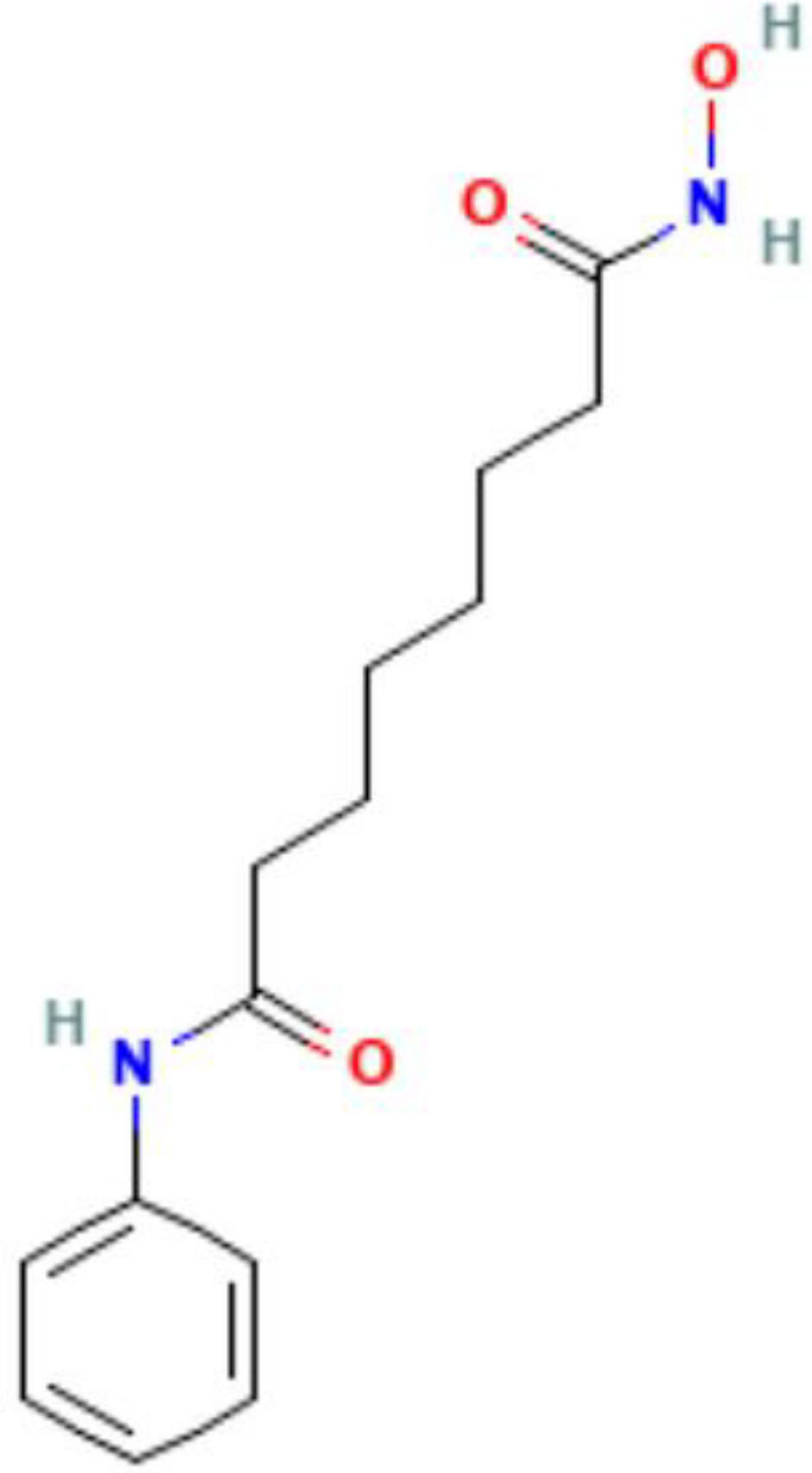		Advanced Ovarian Carcinoma	NCT00976183
				Olaparib	Breast Cancer	NCT03742245
				Pembrolizumab	NSCLC	NCT02638090
		Givinostat (ITF2357)	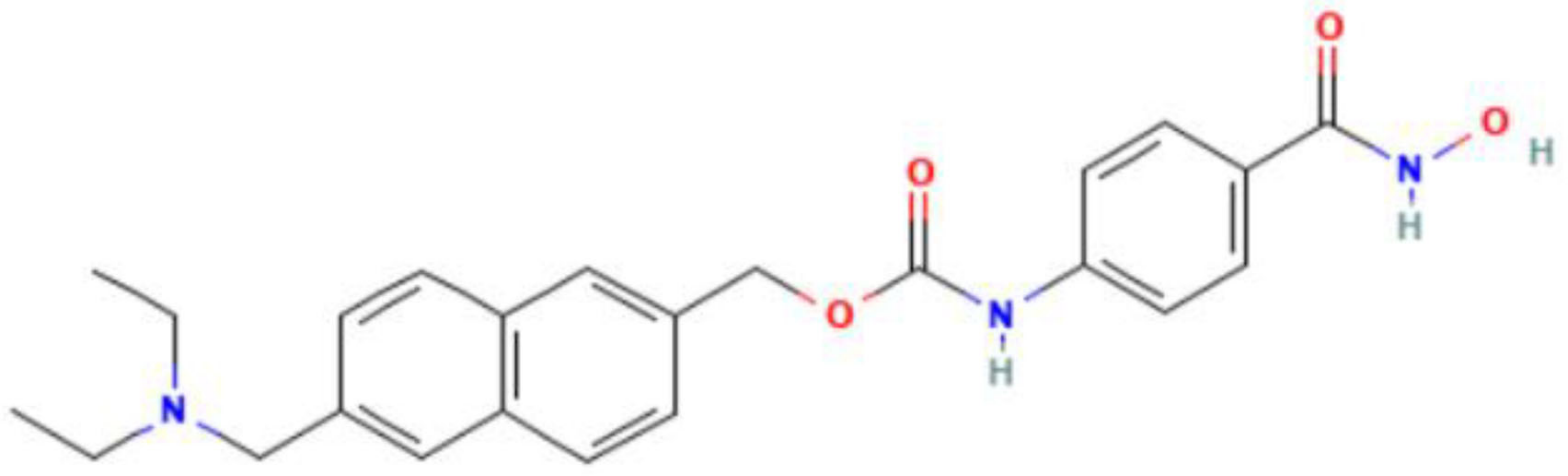		Systemic Juvenile Idiopathic Arthritis	NCT00570661
					Hodgkin's Lymphoma	NCT00496431
		Mocetinostat (MGCD0103)	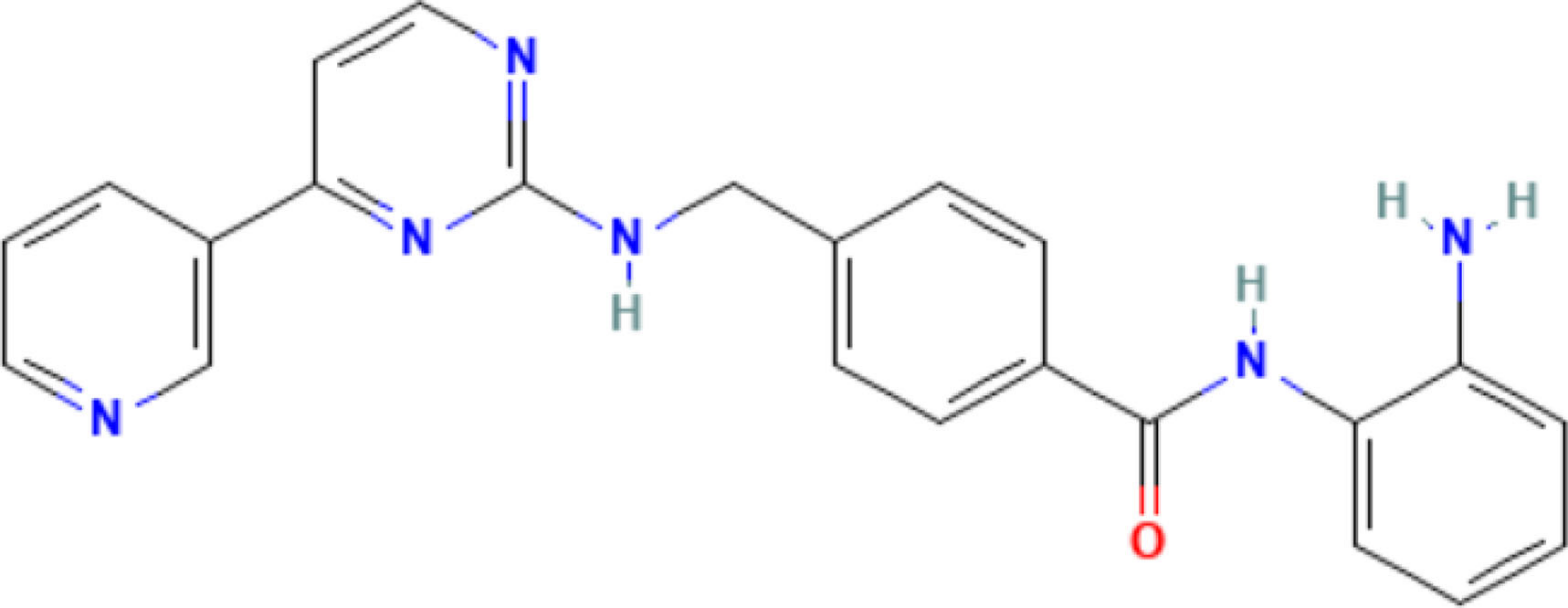		Diffuse Large B-Cell Lymphoma and Follicular Lymphoma	NCT02282358
					Rhabdomyo-sarcoma	NCT04299113
				Pembrolizumab	Advanced Lung Cancer	NCT03220477
				Brentuximab vedotin	Hodgkin Lymphoma	NCT02429375
				Durvalumab	Advanced Solid Tumors and NSCLC	NCT02805660
				Durvalumab	Squamous Cell Carcinoma of the Oral Cavity	NCT02993991
		Tucidinostat (Chidamide)	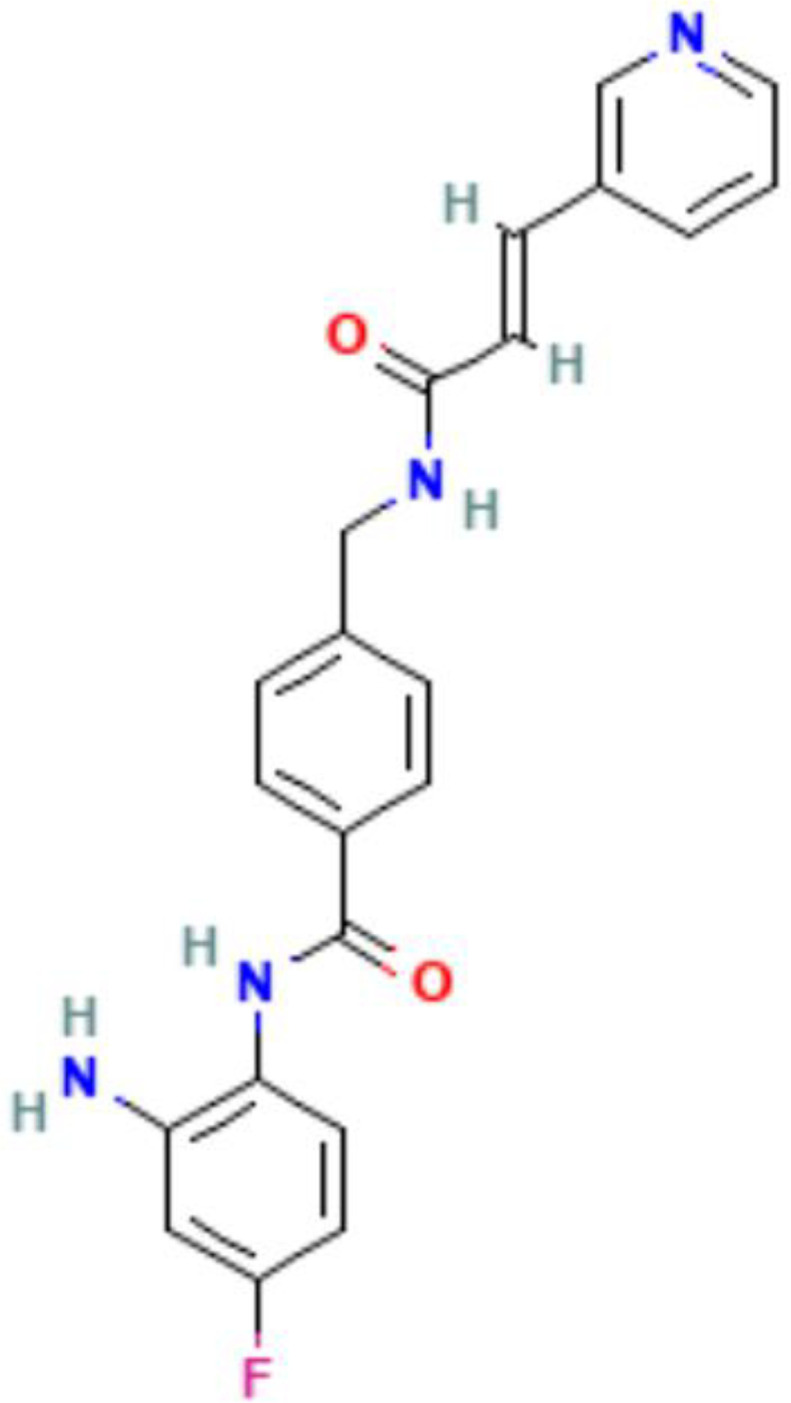		Breast Cancer	NCT05276713
				Sintilimab	Neuroendocrine Neoplasm	NCT05113355
				Apatinib	Advanced Osteosarcoma	NCT06125171
				Zimberelimab	Metastatic Triple-negative Breast Cancer	NCT05632848
				Toripalimab + Bevacizumab	Esophagus Cancer, Gastric Cancer	NCT05163483
				Toripalimab	Advanced Cervical Cancer	NCT04651127
				Tislelizumab	Advanced NSCLC	NCT05519865
		Belinostat (PXD101)	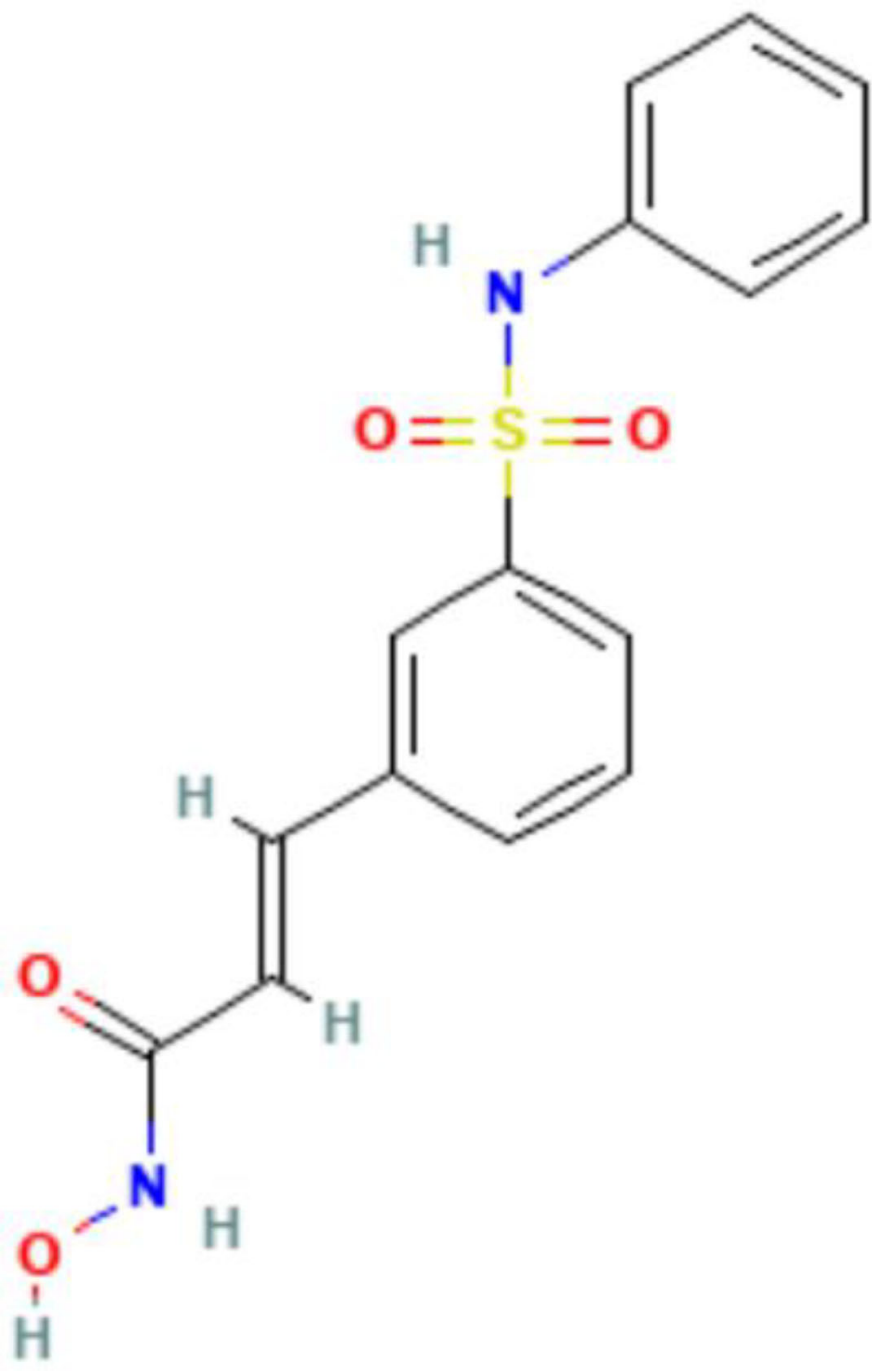		Ovarian Cancer	NCT00421889
Histone methylation	EZH2 inhibitor	Tazemetostat	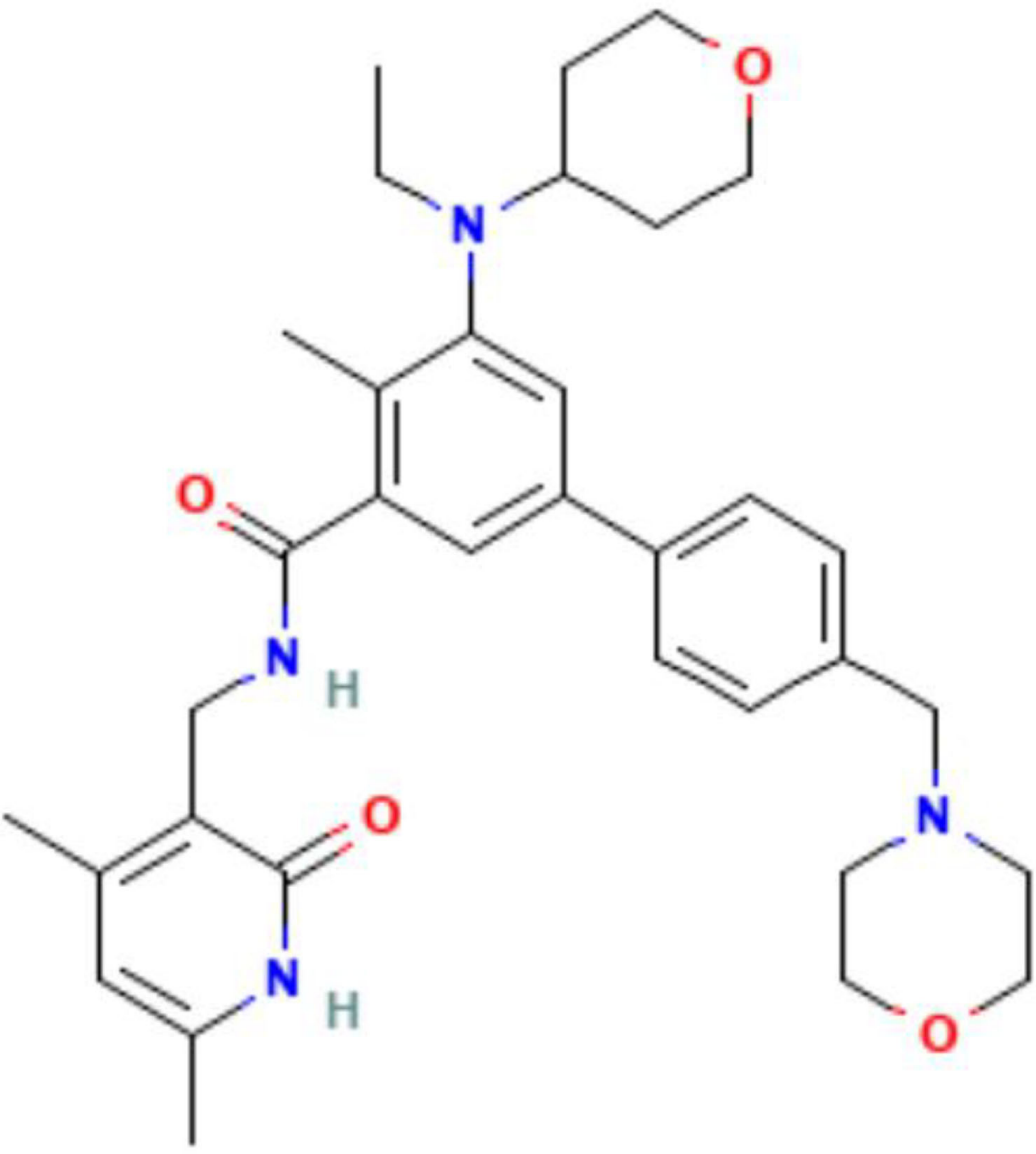		Malignant Peripheral Nerve Sheath Tumors	NCT04917042
					NSCLC	NCT05467748
					Malignant Mesothelioma	NCT02860286
					B-cell Non-Hodgkin's Lymphoma	NCT03009344
					Recurrent Ovarian or Endometrial Cancer	NCT03348631
DNA methylation	DNMT inhibitor	Guadecitabine (SGI-110)	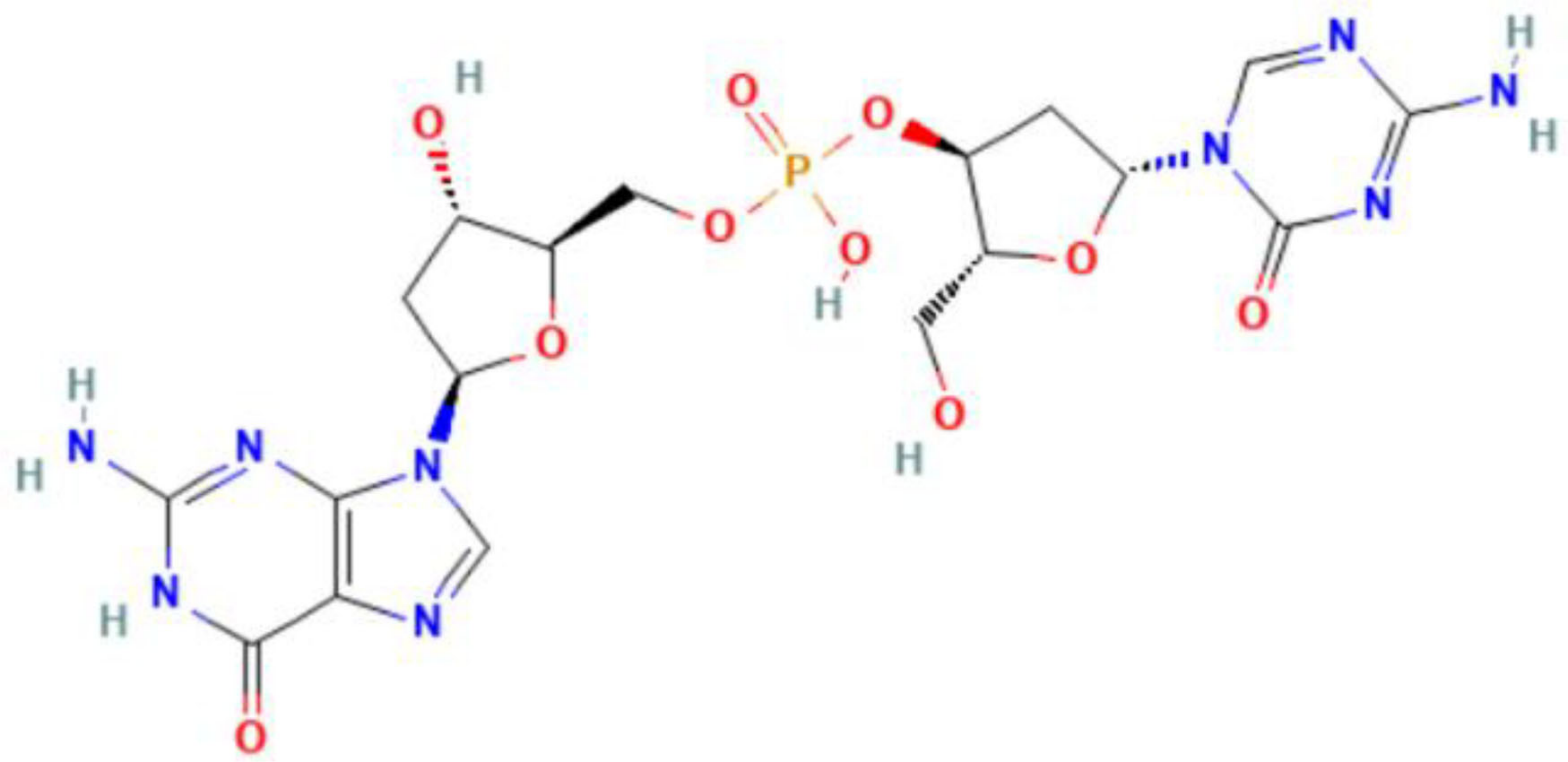	Ipilimumab	Unresectable or Metastatic Melanoma	NCT02608437
				Pembrolizumab	Refractory Solid Tumours	NCT02998567
					Ovarian, Primary Peritoneal, or Fallopian Tube Cancer	NCT02901899
					Advanced Lung Cancer	NCT03220477
				Atezolizumab	Refractory or Resistant Urothelial Carcinoma	NCT03179943
				Ipilimumab + Nivolumab	Melanoma and NSCLC	NCT04250246
		Decitabine	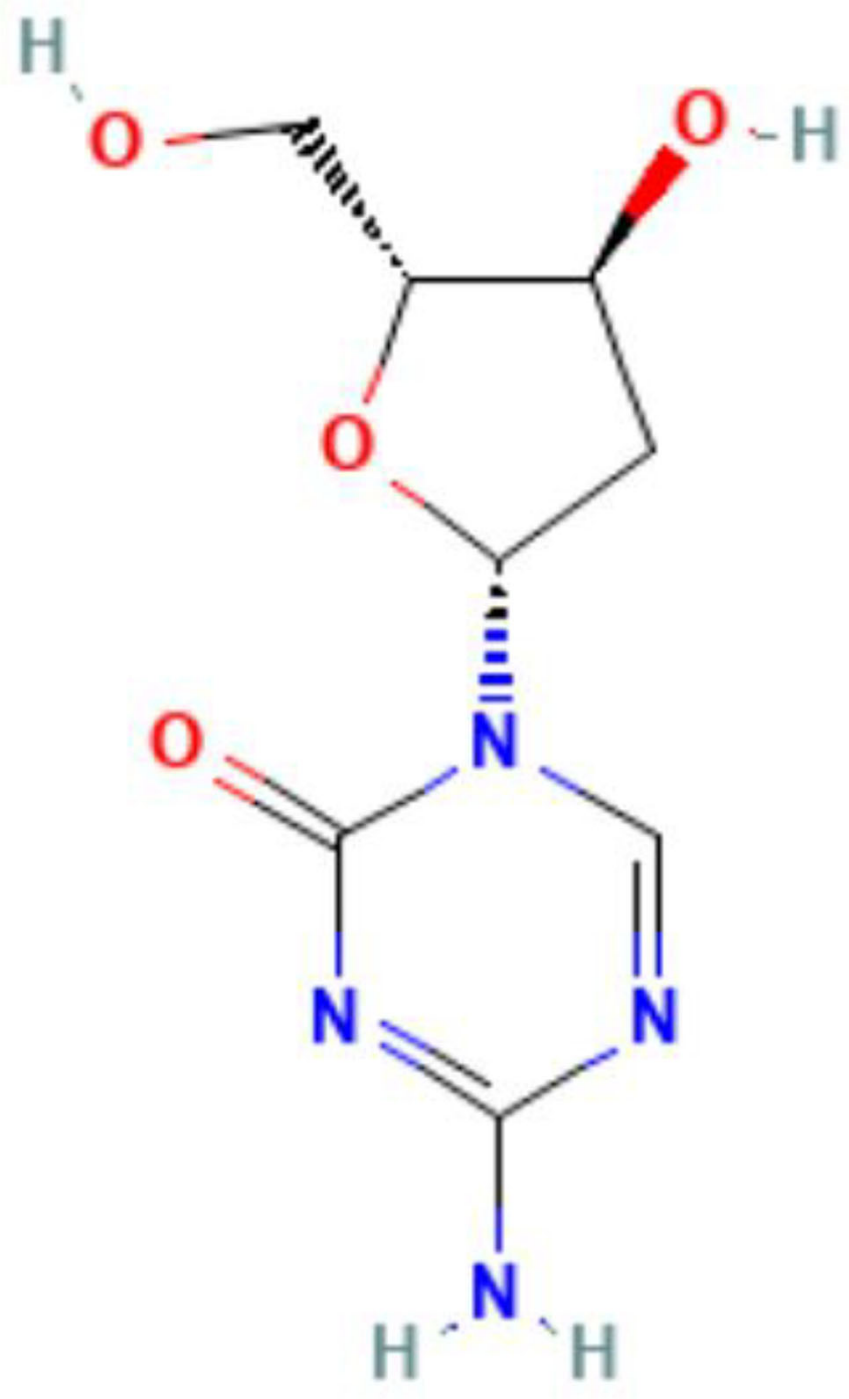		ITP	NCT01568333
				Pembrolizumab	MDS or AML	NCT03969446
				Pembrolizumab	NSCLC and Esophageal Carcinomas	NCT03233724
				DEC-205/​NY-ESO-1 Fusion Protein CDX-1401 and Poly ICLC	MDS or AML	NCT01834248
				TQB2450 Injection (PD-L1 Monoclonal Antibody)	Digestive System Tumors	NCT04611711
				Sintilimab	NK/T-cell Lymphoma	NCT04279379
		Azacitidine	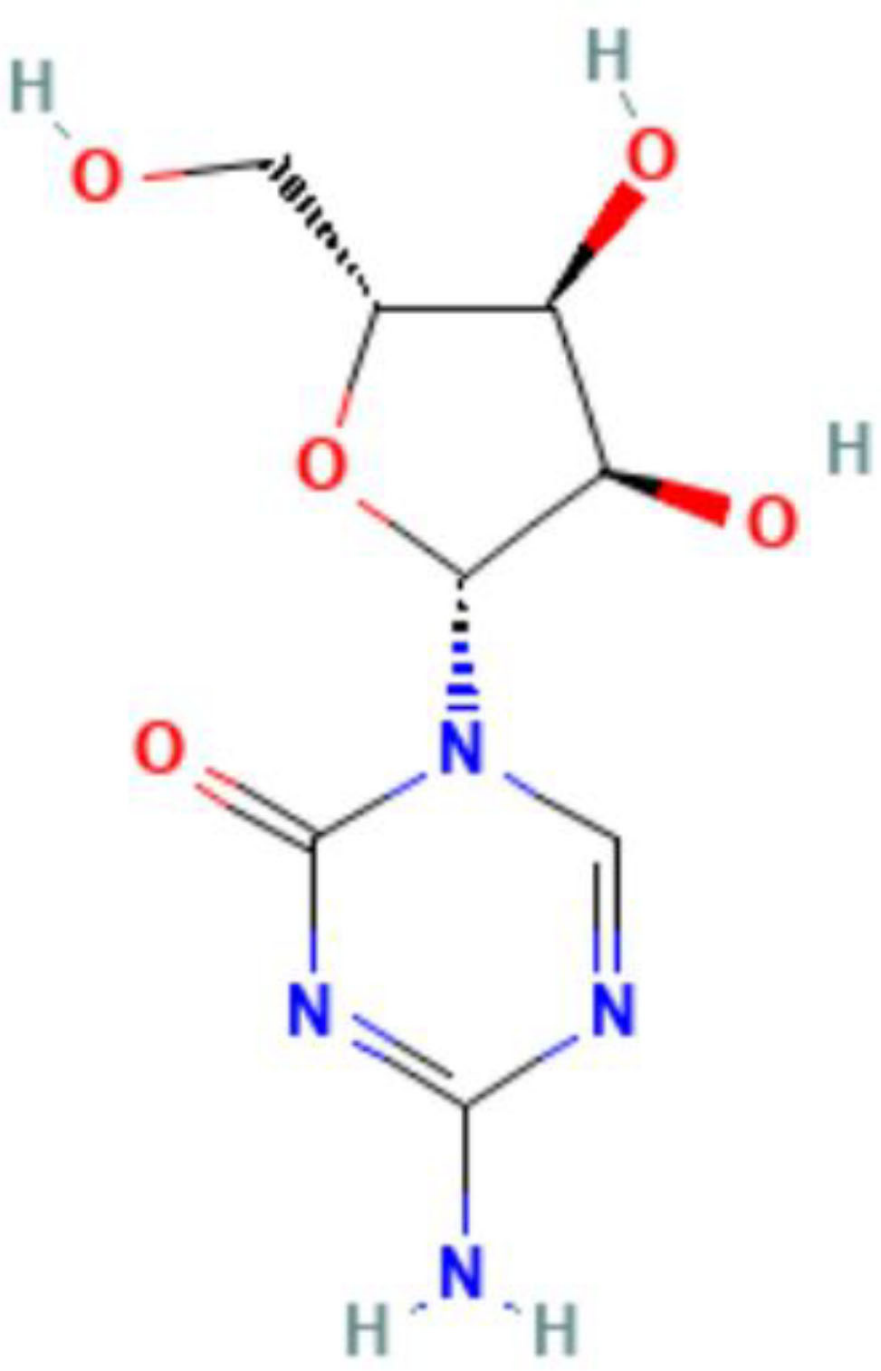		Systemic Auto-immune and Inflammatory Disorders	NCT02985190
				Pembrolizumab	Ovarian cancer	NCT02900560
				Pembrolizumab	AML	NCT02845297
				Pembrolizumab		NCT03769532
				Nivolumab		NCT03825367
				Nivolumab + Relatlimab		NCT04913922
				Ipilimumab/ Nivolumab		NCT02397720
				Pembrolizumab	Pancreatic Cancer	NCT03264404
				Avelumab + Utomilumab + Rituximab	Diffuse Large B-cell Lymphoma	NCT02951156

AML, Acute Myeloid Leukemia; HDAC, histone deacetylase; DNMT, DNA methyltransferase; MDS, Myelodysplastic Syndrome; NSCLC, Non-small cell lung cancer; ITP, Immune Thrombocytopenia.

Images of chemical structure from PubChem website.

## 5 Discussion

Although advances in detection technology have provided a profound understanding that autoimmune diseases may indeed be influenced by genetic and environmental factors to some extent, a growing body of research has suggested that epigenetics also play a nonnegligible role. The theory of epigenetics has provided a new perspective for decoding the secrets of autoimmune diseases and has facilitated a substantial progress in exploring the mechanisms and characteristics of these diseases providing novel biomarkers and potential treatments (65). Epigenetic modifications are altered in patients with autoimmune diseases through a variety of mechanisms due to immune system disorders. Using high-throughput techniques to characterize these and the changes in the content or function of modified enzymes is beneficial for accurately identifying targets that affect the pathogenesis and progression of autoimmune diseases (231).

Despite significant advancements in the field of epigenetics, several crucial issues remain unresolved. For example, both autoimmune diseases and epigenetic modifications demonstrate considerable heterogeneity. Different diseases have varying manifestations, and even the same disease can present distinct clinical manifestations at different stages; consequently, this complexity poses significant difficulties in developing effective treatment strategies (232). Given the dynamic and reversible nature of epigenetic modifications that involve interactions with various enzymes and molecules, a promising approach for treating autoimmune diseases involves targeting and modulating these aberrant modifications. Various epigenetic drugs, such as DNMT and HDAC inhibitors, have been extensively used in cancer treatment, showing good efficacy (233). Autoimmune diseases and cancer share many similarities in their etiology, and immune-related treatments are widely used for both; therefore, extensive research has been focused on the use of epigenetic drugs for treating autoimmune diseases, as these drugs are highly targeted and may have fewer side effects than traditional immunosuppressants. Moreover, the development of more efficient combination therapies based on the epigenetic theory is an exciting prospect, which will bring great benefits to patients with autoimmune diseases (234). However, clinical trials using epigenetic drugs to treat autoimmune diseases are relatively rare, and their efficacy is unclear. As epigenetic drugs may exert genome-wide effects, specifically targeting a gene without causing systemic effects is a considerable challenge. Due to the extensive heterogeneity of diseases and complexity of the immune system, various significant challenges remain in the development and production of more targeted and high-affinity therapeutic drugs. Further research and trials are needed to generate convincing evidence to further improve epigenetic therapies.

In summary, this review described several epigenetic mechanisms, highlighting the close relationship and interactions between epigenetics and autoimmune diseases, and providing new insights into the use of epigenetic modifications and their modifying enzymes as biomarkers and therapeutic targets for treating autoimmune diseases. Although not discussed in this article, the role of ncRNAs in autoimmune diseases has been well described (235). Given the current heavy burden on patients with autoimmune diseases worldwide, more in-depth collaborative research is urgently required in the fields of epigenetics and autoimmune diseases, which could provide more efficient and personalized therapies for patients with autoimmune diseases.

## Glossary

A: adenine
AIRE: Autoimmune regulator
ATP: Adenosine triphosphate
ALKBH: AlkB homologue
APS-1: Autoimmune polyendocrine syndrome type 1
BCR: B cell receptor
BS: bisulfite sequencing
C: cytosine
cDNA: complementary DNA
CpG: cytosineguanine dinucleotides
CHD: chromodomain helicase DNA-binding
ChIP: chromatin immunoprecipitation
ChIP-seq: Chromatin immunoprecipitation-sequencing
CRP: C-reactive protein
DNMT: DNA methyltransferase
DMF: Dimethyl fumarate
DOT1L: telomeric silencing 1-like
EAE: experimental autoimmune encephalomyelitis
EBV: Epstein-Barr virus
EZH2: Enhancer of zeste homolog 2
FLSs: fibroblast-like synoviocytes
Foxp3: forkhead box P3
FTO: fat-mass and obesity-associated protein
G: guanine
GC: germinal center
GWAS: Genome-wide association studies
HATs: histone acetyltransferases
HDACs: histone deacetylases
HDM: histone demethylase
HMT: histone methyltransferase
HNRNP: heterogeneous nuclear ribonucleoprotein
H3K27ac: acetylated lysine 27 of histone 3
H3K27me3: trimethylated lysine 27 of histone H3
H3K4me3: trimethylated lysine 4 of histone H3
H3K79: lysine 79 of histone H3
H3K9: lysine 9 of histone H3
IBD: inflammatory bowel disease
IFI44L: Interferon-induced protein 44-like
IGF2BP: Insulin-like growth factor-2 mRNA-binding proteins
IL: Interleukin
IFN-γ: interferon-γ
INO80: inositol requiring 80
IPD: inter-pulse duration
IPEX: immunodysregulation polyendocrinopathy enteropathy X-linked
ISWI: imitation switch
KATs: lysine acetyltransferases
LN: lupus nephritis
LSD: Lysine-specific demethylase
MBD: methyl-CpG binding domain
METTL4: Methyltransferase like 4
MHC: Major Histocompatibility Complex
MS: multiple sclerosis
MSG: minor salivary gland
MX1: myxoma resistance protein 1
MYST: MOZ, Ybf2/Sas3, Sas2, Tip60
mtDNA: mitochondrial DNA
mTOR: mammalian target of rapamycin
mTORC1: mechanistic target of rapamycin complex 1
m1A: N1-methyladenosine
4mC: N4-methyldeoxycytosine
m6A: N6-methyladenosine
6mA: N6-methyldeoxyadenosine
m6Am: N6,2’-O-dimethyladenosine
m7G: N7-methylguanosine
mTECs: medullary thymic epithelial cells
NAD: nicotinamide adenine dinucleotide
NGS: next-generation sequencing
NSAIDs: non-steroidal anti-inflammatory drugs
NuRD: nuclesome remodeling deactylase
nTreg: natural Treg
PBMCs: peripheral blood mononuclear cells
PTGs: peripheral tissue genes
PHD: plant homeodomain
PBRM1: polybromo-1
PCR: polymerase chain reaction
PRMT: protein arginine methyltransferase
pSS: primary Sjögren syndrome
Ψ: pseudouridine
QC: quality control
RA: rheumatoid arthritis
RASFs: RA synovial fibroblasts
RLRs: retinoic acid-inducible gene-I-like receptors
RORγt: retinoic acid-related orphan receptor γt
RT: reverse transcription
SAM: S-adenosyl methionine
7βS: seven-beta-strand domain
SEs: super enhancers
SFs: synovial fibroblasts
SLE: systemic lupus erythematosus
SLEDAI: SLE disease activity index
SMRT-seq: Single-molecule real-time sequencing
SPEN: Split ends homolog
SS: Sjögren’s Syndrome
SSBP1: single-stranded DNA-binding protein 1
SSc: systemic sclerosis
SWI/SNF: switching defective/sucrose nonfermenting
T: thymine
TCR: T cell receptor
TET: Ten-eleven translocation
Th17: T helper 17
TLR7: Toll-like receptor 7
TNFAIP3: TNF-α-induced protein 3
tRNA: transfer RNA
Treg: regulatory T cells
T1D: diabetes mellitus type 1
U: uracil
UC: ulcerative colitis
WGBS: whole-genome bisulfite sequencing
WTAP: Wilms tumor 1-associating protein
XCI: X chromosome inactivation
YTH: YT521-B homology
YTHDC: YTH domain-containing protein
YTHDF: YTH domain family
ZMWs: zero-mode waveguides
3’UTR: 3’
5mC: 5-methylcytosine
5hmC: 5-hydroxymethylcytosine
5mC: 5-methylcytosine
